# The impact of viral mutations on recognition by SARS-CoV-2 specific T cells

**DOI:** 10.1016/j.isci.2021.103353

**Published:** 2021-10-28

**Authors:** Thushan I. de Silva, Guihai Liu, Benjamin B. Lindsey, Danning Dong, Shona C. Moore, Nienyun Sharon Hsu, Dhruv Shah, Dannielle Wellington, Alexander J. Mentzer, Adrienn Angyal, Rebecca Brown, Matthew D. Parker, Zixi Ying, Xuan Yao, Lance Turtle, Susanna Dunachie, COVID-19 Genomics UK (COG-UK) Consortium, David M. Aanensen, Khalil Abudahab, Helen Adams, Alexander Adams, Safiah Afifi, Dinesh Aggarwal, Shazaad S.Y. Ahmad, Louise Aigrain, Adela Alcolea-Medina, Nabil-Fareed Alikhan, Elias Allara, Roberto Amato, Tara Annett, Stephen Aplin, Cristina V. Ariani, Hibo Asad, Amy Ash, Paula Ashfield, Fiona Ashford, Laura Atkinson, Stephen W. Attwood, Cressida Auckland, Alp Aydin, David J. Baker, Paul Baker, Carlos E. Balcazar, Jonathan Ball, Jeffrey C. Barrett, Magdalena Barrow, Edward Barton, Matthew Bashton, Andrew R. Bassett, Rahul Batra, Chris Baxter, Nadua Bayzid, Charlotte Beaver, Angela H. Beckett, Shaun M. Beckwith, Luke Bedford, Robert Beer, Andrew Beggs, Katherine L. Bellis, Louise Berry, Beatrice Bertolusso, Angus Best, Emma Betteridge, David Bibby, Kelly Bicknell, Debbie Binns, Alec Birchley, Paul W. Bird, Chloe Bishop, Rachel Blacow, Victoria Blakey, Beth Blane, Frances Bolt, James Bonfield, Stephen Bonner, David Bonsall, Tim Boswell, Andrew Bosworth, Yann Bourgeois, Olivia Boyd, Declan T. Bradley, Cassie Breen, Catherine Bresner, Judith Breuer, Stephen Bridgett, Iraad F. Bronner, Ellena Brooks, Alice Broos, Julianne R. Brown, Giselda Bucca, Sarah L. Buchan, David Buck, Matthew Bull, Phillipa J. Burns, Shirelle Burton-Fanning, Timothy Byaruhanga, Matthew Byott, Sharon Campbell, Alessandro M. Carabelli, James S. Cargill, Matthew Carlile, Silvia F. Carvalho, Anna Casey, Anibolina Castigador, Jana Catalan, Vicki Chalker, Nicola J. Chaloner, Meera Chand, Joseph G. Chappell, Themoula Charalampous, Wendy Chatterton, Yasmin Chaudhry, Carol M. Churcher, Gemma Clark, Phillip Clarke, Benjamin J. Cogger, Kevin Cole, Jennifer Collins, Rachel Colquhoun, Thomas R. Connor, Kate F. Cook, Jason Coombes, Sally Corden, Claire Cormie, Nicholas Cortes, Marius Cotic, Seb Cotton, Simon Cottrell, Lindsay Coupland, MacGregor Cox, Alison Cox, Noel Craine, Liam Crawford, Aidan Cross, Matthew R. Crown, Dorian Crudgington, Nicola Cumley, Tanya Curran, Martin D. Curran, Ana da Silva Filipe, Gavin Dabrera, Alistair C. Darby, Rose K. Davidson, Alisha Davies, Robert M. Davies, Thomas Davis, Daniela de Angelis, Elen De Lacy, Leonardo de Oliveira Martins, Johnny Debebe, Rebecca Denton-Smith, Samir Dervisevic, Rebecca Dewar, Jayasree Dey, Joana Dias, Donald Dobie, Matthew J. Dorman, Fatima Downing, Megan Driscoll, Louis du Plessis, Nichola Duckworth, Jillian Durham, Kirstine Eastick, Lisa J. Easton, Richard Eccles, Jonathan Edgeworth, Sue Edwards, Kate El Bouzidi, Sahar Eldirdiri, Nicholas Ellaby, Scott Elliott, Gary Eltringham, Leah Ensell, Michelle J. Erkiert, Marina Escalera Zamudio, Sarah Essex, Johnathan M. Evans, Cariad Evans, William Everson, Derek J. Fairley, Karlie Fallon, Arezou Fanaie, Ben W. Farr, Christopher Fearn, Theresa Feltwell, Lynne Ferguson, Laia Fina, Flavia Flaviani, Vicki M. Fleming, Sally Forrest, Ebenezer Foster-Nyarko, Benjamin H. Foulkes, Luke Foulser, Mireille Fragakis, Dan Frampton, Sarah Francois, Christophe Fraser, Timothy M. Freeman, Helen Fryer, Marc Fuchs, William Fuller, Kavitha Gajee, Katerina Galai, Abbie Gallagher, Eileen Gallagher, Michael D. Gallagher, Marta Gallis, Amy Gaskin, Bree Gatica-Wilcox, Lily Geidelberg, Matthew Gemmell, Iliana Georgana, Ryan P. George, Laura Gifford, Lauren Gilbert, Sophia T. Girgis, Sharon Glaysher, Emily J. Goldstein, Tanya Golubchik, Andrea N. Gomes, Sónia Gonçalves, Ian G. Goodfellow, Scott Goodwin, Salman Goudarzi, Marina Gourtovaia, Clive Graham, Lee Graham, Paul R. Grant, Luke R. Green, Angie Green, Jane Greenaway, Richard Gregory, Martyn Guest, Rory N. Gunson, Ravi K. Gupta, Bernardo Gutierrez, Sam T. Haldenby, William L. Hamilton, Samantha E. Hansford, Tanzina Haque, Kathryn A. Harris, Ian Harrison, Ewan M. Harrison, Jennifer Hart, John A. Hartley, William T. Harvey, Matthew Harvey, Mohammed O. Hassan-Ibrahim, Judith Heaney, Thomas Helmer, John H. Henderson, Andrew R. Hesketh, Jessica Hey, David Heyburn, Ellen E. Higginson, Verity Hill, Jack D. Hill, Rachel A. Hilson, Ember Hilvers, Matthew T.G. Holden, Amy Hollis, Christopher W. Holmes, Nadine Holmes, Alison H. Holmes, Richard Hopes, Hailey R. Hornsby, Myra Hosmillo, Catherine Houlihan, Hannah C. Howson-Wells, Jonathan Hubb, Hannah Huckson, Warwick Hughes, Joseph Hughes, Margaret Hughes, Stephanie Hutchings, Giles Idle, Chris J. Illingworth, Robert Impey, Dianne Irish-Tavares, Miren Iturriza-Gomara, Rhys Izuagbe, Chris Jackson, Ben Jackson, Leigh M. Jackson, Kathryn A. Jackson, David K. Jackson, Aminu S. Jahun, Victoria James, Keith James, Christopher Jeanes, Aaron R. Jeffries, Sarah Jeremiah, Andrew Jermy, Michaela John, Rob Johnson, Kate Johnson, Ian Johnston, Owen Jones, Sophie Jones, Hannah Jones, Christopher R. Jones, Neil Jones, Amelia Joseph, Sarah Judges, Gemma L. Kay, Sally Kay, Jon-Paul Keatley, Alexander J. Keeley, Anita Kenyon, Leanne M. Kermack, Manjinder Khakh, Stephen P. Kidd, Maimuna Kimuli, Stuart Kirk, Christine Kitchen, Katie Kitchman, Bridget A. Knight, Cherian Koshy, Moritz U.G. Kraemer, Sara Kumziene-Summerhayes, Dominic Kwiatkowski, Angie Lackenby, Kenneth G. Laing, Temi Lampejo, Cordelia F. Langford, Deborah Lavin, Andrew I. Lawton, Jack Lee, David Lee, Stefanie V. Lensing, Steven Leonard, Lisa J. Levett, Thanh Le-Viet, Jonathan Lewis, Kevin Lewis, Jennifier Liddle, Steven Liggett, Patrick J. Lillie, Michelle M. Lister, Rich Livett, Stephanie Lo, Nicholas J. Loman, Matthew W. Loose, Stavroula F. Louka, Katie F. Loveson, Sarah Lowdon, Hannah Lowe, Helen L. Lowe, Anita O. Lucaci, Catherine Ludden, Jessica Lynch, Ronan A. Lyons, Katrina Lythgoe, Nicholas W. Machin, George MacIntyre-Cockett, Andrew Mack, Ben Macklin, Alasdair Maclean, Emily Macnaughton, Pinglawathee Madona, Mailis Maes, Laurentiu Maftei, Adhyana I.K. Mahanama, Tabitha W. Mahungu, Daniel Mair, Joshua Maksimovic, Cassandra S. Malone, Daniel Maloney, Nikos Manesis, Robin Manley, Anna Mantzouratou, Angela Marchbank, Arun Mariappan, Inigo Martincorena, Rocio T. Martinez Nunez, Alison E. Mather, Patrick Maxwell, Megan Mayhew, Tamyo Mbisa, Clare M. McCann, Shane A. McCarthy, Kathryn McCluggage, Patrick C. McClure, J.T. McCrone, Martin P. McHugh, James P. McKenna, Caoimhe McKerr, Georgina M. McManus, Claire L. McMurray, Claire McMurray, Alan McNally, Lizzie Meadows, Nathan Medd, Oliver Megram, Mirko Menegazzo, Ian Merrick, Stephen L. Michell, Michelle L. Michelsen, Mariyam Mirfenderesky, Jeremy Mirza, Julia Miskelly, Emma Moles-Garcia, Robin J. Moll, Zoltan Molnar, Irene M. Monahan, Matteo Mondani, Siddharth Mookerjee, Christopher Moore, Jonathan Moore, Nathan Moore, Catherine Moore, Helen Morcrette, Sian Morgan, Mari Morgan, Matilde Mori, Arthur Morriss, Samuel Moses, Craig Mower, Peter Muir, Afrida Mukaddas, Florence Munemo, Robert Munn, Abigail Murray, Leanne J. Murray, Darren R. Murray, Manasa Mutingwende, Richard Myers, Eleni Nastouli, Gaia Nebbia, Andrew Nelson, Charlotte Nelson, Sam Nicholls, Jenna Nichols, Roberto Nicodemi, Kyriaki Nomikou, Justin O’Grady, Sarah O'Brien, Mina Odedra, Natasha Ohemeng-Kumi, Karen Oliver, Richard J. Orton, Husam Osman, xeine O'Toole, Nicole Pacchiarini, Debra Padgett, Andrew J. Page, Emily J. Park, Naomi R. Park, Surendra Parmar, David G. Partridge, David Pascall, Amita Patel, Bindi Patel, Steve Paterson, Brendan A.I. Payne, Sharon J. Peacock, Clare Pearson, Emanuela Pelosi, Benita Percival, Jon Perkins, Malorie Perry, Malte L. Pinckert, Steven Platt, Olga Podplomyk, Manoj Pohare, Marcus Pond, Cassie F. Pope, Radoslaw Poplawski, Jessica Powell, Jennifer Poyner, Liam Prestwood, Anna Price, James R. Price, Jacqui A. Prieto, David T. Pritchard, Sophie J. Prosolek, Georgia Pugh, Monika Pusok, Oliver G. Pybus, Hannah M. Pymont, Michael A. Quail, Joshua Quick, Clara Radulescu, Jayna Raghwani, Manon Ragonnet-Cronin, Lucille Rainbow, Diana Rajan, Shavanthi Rajatileka, Newara A. Ramadan, Andrew Rambaut, John Ramble, Paul A. Randell, Paul Randell, Liz Ratcliffe, Veena Raviprakash, Mohammad Raza, Nicholas M. Redshaw, Sara Rey, Nicola Reynolds, Alex Richter, David L. Robertson, Esther Robinson, Samuel C. Robson, Fiona Rogan, Stefan Rooke, Will Rowe, Sunando Roy, Steven Rudder, Chris Ruis, Steven Rushton, Felicity Ryan, Kordo Saeed, Buddhini Samaraweera, Christine M. Sambles, Roy Sanderson, Theo Sanderson, Fei Sang, Thea Sass, Emily Scher, Garren Scott, Carol Scott, Jasveen Sehmi, Sharif Shaaban, Divya Shah, Jessica Shaw, Ekaterina Shelest, James G. Shepherd, Liz A. Sheridan, Nicola Sheriff, Lesley Shirley, John Sillitoe, Siona Silviera, David A. Simpson, Aditi Singh, Dawn Singleton, Timofey Skvortsov, Tim J. Sloan, Graciela Sluga, Ken Smith, Kim S. Smith, Perminder Smith, Darren L. Smith, Louise Smith, Colin P. Smith, Nikki Smith, Katherine L. Smollett, Luke B. Snell, Thomas Somassa, Joel Southgate, Karla Spellman, Michael H. Spencer Chapman, Lewis G. Spurgin, Moira J. Spyer, Rachael Stanley, William Stanley, Thomas D. Stanton, Igor Starinskij, Joanne Stockton, Susanne Stonehouse, Nathaniel Storey, David J. Studholme, Malur Sudhanva, Emma Swindells, Yusri Taha, Ngee Keong Tan, Julian W. Tang, Miao Tang, Ben E.W. Taylor, Joshua F. Taylor, Sarah Taylor, Ben Temperton, Kate E. Templeton, Claire Thomas, Laura Thomson, Emma C. Thomson, Alicia Thornton, Scott A.J. Thurston, John A. Todd, Rachael Tomb, Lily Tong, Gerry Tonkin-Hill, M. Estee Torok, Jaime M. Tovar-Corona, Amy Trebes, Alexander J. Trotter, Ioulia Tsatsani, Robyn Turnbull, Katherine A. Twohig, Helen Umpleby, Anthony P. Underwood, Edith E. Vamos, Tetyana I. Vasylyeva, Sreenu Vattipally, Gabrielle Vernet, Barry B. Vipond, Erik M. Volz, Sarah Walsh, Dennis Wang, Ben Warne, Joanna Warwick-Dugdale, Elizabeth Wastnedge, Joanne Watkins, Louisa K. Watson, Sheila Waugh, Hermione J. Webster, Danni Weldon, Elaine Westwick, Thomas Whalley, Helen Wheeler, Mark Whitehead, Max Whiteley, Andrew Whitwham, Claudia Wierzbicki, Nicholas J. Willford, Lesley-Anne Williams, Rebecca Williams, Cheryl Williams, Chris Williams, Charlotte A. Williams, Rachel J. Williams, Thomas Williams, Catryn Williams, Kathleen A. Williamson, Eleri Wilson-Davies, Eric Witele, Karen T. Withell, Adam A. Witney, Paige Wolverson, Nick Wong, Trudy Workman, Victoria Wright, Derek W. Wright, Tim Wyatt, Sarah Wyllie, Li Xu-McCrae, Mehmet Yavus, Geraldine Yaze, Corin A. Yeats, Gonzalo Yebra, Wen C. Yew, Gregory R. Young, Jamie Young, Alex E. Zarebski, Peijun Zhang, Mala K. Maini, Graham Ogg, Julian C. Knight, ISARIC4C Investigators, J. Kenneth Baillie, Malcolm G. Semple, Peter J.M. Openshaw, Gail Carson, Beatrice Alex, Petros Andrikopoulos, Benjamin Bach, Wendy S. Barclay, Debby Bogaert, Meera Chand, Kanta Chechi, Graham S. Cooke, Ana da Silva Filipe, Annemarie B. Docherty, Gonçalo dos Santos Correia, Marc-Emmanuel Dumas, Jake Dunning, Tom Fletcher, Christopher A. Green, William Greenhalf, Julian L. Griffin, Rishi K. Gupta, Ewen M. Harrison, Julian A. Hiscox, Antonia Ying Wai Ho, Peter W. Horby, Samreen Ijaz, Saye Khoo, Paul Klenerman, Andrew Law, Matthew R. Lewis, Sonia Liggi, Wei Shen Lim, Lynn Maslen, Alexander J. Mentzer, Laura Merson, Alison M. Meynert, Mahdad Noursadeghi, Michael Olanipekun, Anthonia Osagie, Massimo Palmarini, Carlo Palmieri, William A. Paxton, Georgios Pollakis, Nicholas Price, Andrew Rambaut, David L. Robertson, Clark D. Russell, Vanessa Sancho-Shimizu, Caroline J. Sands, Janet T. Scott, Louise Sigfrid, Tom Solomon, Shiranee Sriskandan, David Stuart, Charlotte Summers, Olivia V. Swann, Zoltan Takats, Panteleimon Takis, Richard S. Tedder, A.A. Roger Thompson, Emma C. Thomson, Ryan S. Thwaites, Maria Zambon, Hayley Hardwick, Chloe Donohue, Fiona Griffiths, Wilna Oosthuyzen, Cara Donegan, Rebecca G. Spencer, Jo Dalton, Michelle Girvan, Egle Saviciute, Stephanie Roberts, Janet Harrison, Laura Marsh, Marie Connor, Sophie Halpin, Clare Jackson, Carrol Gamble, Daniel Plotkin, James Lee, Gary Leeming, Andrew Law, Murray Wham, Sara Clohisey, Ross Hendry, James Scott-Brown, Victoria Shaw, Sarah E. McDonald, Seán Keating, Katie A. Ahmed, Jane A. Armstrong, Milton Ashworth, Innocent G. Asiimwe, Siddharth Bakshi, Samantha L. Barlow, Laura Booth, Benjamin Brennan, Katie Bullock, Benjamin W.A. Catterall, Jordan J. Clark, Emily A. Clarke, Sarah Cole, Louise Cooper, Helen Cox, Christopher Davis, Oslem Dincarslan, Chris Dunn, Philip Dyer, Angela Elliott, Anthony Evans, Lorna Finch, Lewis W.S. Fisher, Terry Foster, Isabel Garcia-Dorival, Philip Gunning, Catherine Hartley, Rebecca L. Jensen, Christopher B. Jones, Trevor R. Jones, Shadia Khandaker, Katharine King, Robyn T. Kiy, Chrysa Koukorava, Annette Lake, Suzannah Lant, Diane Latawiec, Lara Lavelle-Langham, Daniella Lefteri, Lauren Lett, Lucia A. Livoti, Maria Mancini, Sarah McDonald, Laurence McEvoy, John McLauchlan, Soeren Metelmann, Nahida S. Miah, Joanna Middleton, Joyce Mitchell, Shona C. Moore, Ellen G. Murphy, Rebekah Penrice-Randal, Jack Pilgrim, Tessa Prince, Will Reynolds, P. Matthew Ridley, Debby Sales, Victoria E. Shaw, Rebecca K. Shears, Benjamin Small, Krishanthi S. Subramaniam, Agnieska Szemiel, Aislynn Taggart, Jolanta Tanianis-Hughes, Jordan Thomas, Erwan Trochu, Libby van Tonder, Eve Wilcock, J. Eunice Zhang, Lisa Flaherty, Nicole Maziere, Emily Cass, Alejandra Doce Carracedo, Nicola Carlucci, Anthony Holmes, Hannah Massey, Lee Murphy, Nicola Wrobel, Sarah McCafferty, Kirstie Morrice, Alan MacLean, Kayode Adeniji, Daniel Agranoff, Ken Agwuh, Dhiraj Ail, Erin L. Aldera, Ana Alegria, Sam Allen, Brian Angus, Abdul Ashish, Dougal Atkinson, Shahedal Bari, Gavin Barlow, Stella Barnass, Nicholas Barrett, Christopher Bassford, Sneha Basude, David Baxter, Michael Beadsworth, Jolanta Bernatoniene, John Berridge, Colin Berry, Nicola Best, Pieter Bothma, David Chadwick, Robin Brittain-Long, Naomi Bulteel, Tom Burden, Andrew Burtenshaw, Vikki Caruth, David Chadwick, Duncan Chambler, Nigel Chee, Jenny Child, Srikanth Chukkambotla, Tom Clark, Paul Collini, Catherine Cosgrove, Jason Cupitt, Maria-Teresa Cutino-Moguel, Paul Dark, Chris Dawson, Samir Dervisevic, Phil Donnison, Sam Douthwaite, Andrew Drummond, Ingrid DuRand, Ahilanadan Dushianthan, Tristan Dyer, Cariad Evans, Chi Eziefula, Chrisopher Fegan, Adam Finn, Duncan Fullerton, Sanjeev Garg, Sanjeev Garg, Atul Garg, Effrossyni Gkrania-Klotsas, Jo Godden, Arthur Goldsmith, Clive Graham, Elaine Hardy, Stuart Hartshorn, Daniel Harvey, Peter Havalda, Daniel B. Hawcutt, Maria Hobrok, Luke Hodgson, Anil Hormis, Michael Jacobs, Susan Jain, Paul Jennings, Agilan Kaliappan, Vidya Kasipandian, Stephen Kegg, Michael Kelsey, Jason Kendall, Caroline Kerrison, Ian Kerslake, Oliver Koch, Gouri Koduri, George Koshy, Shondipon Laha, Steven Laird, Susan Larkin, Tamas Leiner, Patrick Lillie, James Limb, Vanessa Linnett, Jeff Little, Mark Lyttle, Michael MacMahon, Emily MacNaughton, Ravish Mankregod, Huw Masson, Elijah Matovu, Katherine McCullough, Ruth McEwen, Manjula Meda, Gary Mills, Jane Minton, Mariyam Mirfenderesky, Kavya Mohandas, Quen Mok, James Moon, Elinoor Moore, Patrick Morgan, Craig Morris, Katherine Mortimore, Samuel Moses, Mbiye Mpenge, Rohinton Mulla, Michael Murphy, Megan Nagel, Thapas Nagarajan, Mark Nelson, Lillian Norris, Matthew K. O'Shea, Igor Otahal, Marlies Ostermann, Mark Pais, Carlo Palmieri, Selva Panchatsharam, Danai Papakonstantinou, Hassan Paraiso, Brij Patel, Natalie Pattison, Justin Pepperell, Mark Peters, Mandeep Phull, Stefania Pintus, Jagtur Singh Pooni, Tim Planche, Frank Post, David Price, Rachel Prout, Nikolas Rae, Henrik Reschreiter, Tim Reynolds, Neil Richardson, Mark Roberts, Devender Roberts, Alistair Rose, Guy Rousseau, Bobby Ruge, Brendan Ryan, Taranprit Saluja, Matthias L. Schmid, Aarti Shah, Prad Shanmuga, Anil Sharma, Anna Shawcross, Jeremy Sizer, Manu Shankar-Hari, Richard Smith, Catherine Snelson, Nick Spittle, Nikki Staines, Tom Stambach, Richard Stewart, Pradeep Subudhi, Tamas Szakmany, Kate Tatham, Jo Thomas, Chris Thompson, Robert Thompson, Ascanio Tridente, Darell Tupper-Carey, Mary Twagira, Nick Vallotton, Rama Vancheeswaran, Lisa Vincent-Smith, Shico Visuvanathan, Alan Vuylsteke, Sam Waddy, Rachel Wake, Andrew Walden, Ingeborg Welters, Tony Whitehouse, Paul Whittaker, Ashley Whittington, Padmasayee Papineni, Meme Wijesinghe, Martin Williams, Lawrence Wilson, Sarah Cole, Stephen Winchester, Martin Wiselka, Adam Wolverson, Daniel G. Wootton, Andrew Workman, Bryan Yates, Peter Young, Yanchun Peng, Sarah L. Rowland-Jones, Tao Dong

**Affiliations:** 1 The Florey Institute for Host-Pathogen Interactions and Department of Infection, Immunity and Cardiovascular Disease, Medical School, University of Sheffield, Sheffield S10 2RX, UK; 2 Vaccines and Immunity Theme, Medical Research Council Unit The Gambia at the London School of Hygiene and Tropical Medicine, P.O. Box 273, Banjul, The Gambia; 3 Chinese Academy of Medical Sciences (CAMS) Oxford Institute (COI), University of Oxford, Oxford OX3 7FZ, UK; 4 MRC Human Immunology Unit, MRC Weatherall Institute of Molecular Medicine, Radcliffe Department of Medicine, University of Oxford, Oxford OX3 9DS, UK; 5 Beijing You'an Hospital, Capital Medical University, Beijing, China; 6 CAMS Key Laboratory of Tumor Immunology and Radiation Therapy, Xinjiang Tumor Hospital, Xinjiang Medical University, China; 7 NIHR Health Protection Research Unit in Emerging and Zoonotic Infections, Institute of Infection, Veterinary and Ecological Sciences, University of Liverpool, Liverpool CH64 7TE, UK; 8 Sheffield Bioinformatics Core, The University of Sheffield, Sheffield, UK; 9 Nuffield Department of Medicine, University of Oxford, NDM Research Building, Oxford OX3 7FZ, UK; 10 Wellcome Centre for Human Genetics, University of Oxford, Oxford OX3 7BN, UK; 11 Sheffield Biomedical Research Centre, The University of Sheffield, Sheffield S10 2JF, UK; 12 Tropical & Infectious Disease Unit, Liverpool University Hospitals NHS Foundation Trust (Member of Liverpool Health Partners), Liverpool L7 8XP, UK; 13 Centre For Tropical Medicine and Global Health, Nuffield Department of Clinical Medicine, University of Oxford, Oxford OX3 7LG, UK; 14 Mahidol-Oxford Tropical Medicine Research Unit, Bangkok, Thailand; 15 https://www.cogconsortium.uk; 16 Division of Infection and Immunity, University College London, London WC1E 6BT, UK; 17 https://isaric4c.net

**Keywords:** Phylogenetics, Molecular biology, Immunology, Immune response, Virology

## Abstract

We identify amino acid variants within dominant SARS-CoV-2 T cell epitopes by interrogating global sequence data. Several variants within nucleocapsid and ORF3a epitopes have arisen independently in multiple lineages and result in loss of recognition by epitope-specific T cells assessed by IFN-γ and cytotoxic killing assays. Complete loss of T cell responsiveness was seen due to Q213K in the A∗01:01-restricted CD8+ ORF3a epitope FTSDYYQLY_207-215_; due to P13L, P13S, and P13T in the B∗27:05-restricted CD8+ nucleocapsid epitope QRNAPRITF_9-17_; and due to T362I and P365S in the A∗03:01/A∗11:01-restricted CD8+ nucleocapsid epitope KTFPPTEPK_361-369_. CD8+ T cell lines unable to recognize variant epitopes have diverse T cell receptor repertoires. These data demonstrate the potential for T cell evasion and highlight the need for ongoing surveillance for variants capable of escaping T cell as well as humoral immunity.

## Highlights

• Amino acid variants in dominant SARS-CoV-2 T cell epitopes result in recognition loss
• CD8+ clones with diverse T cell receptor repertoires fail to recognize variant epitopes
• Ongoing surveillance for SARS-CoV-2 variants resulting in T cell evasion is important

## Graphical abstract

**Figure:**
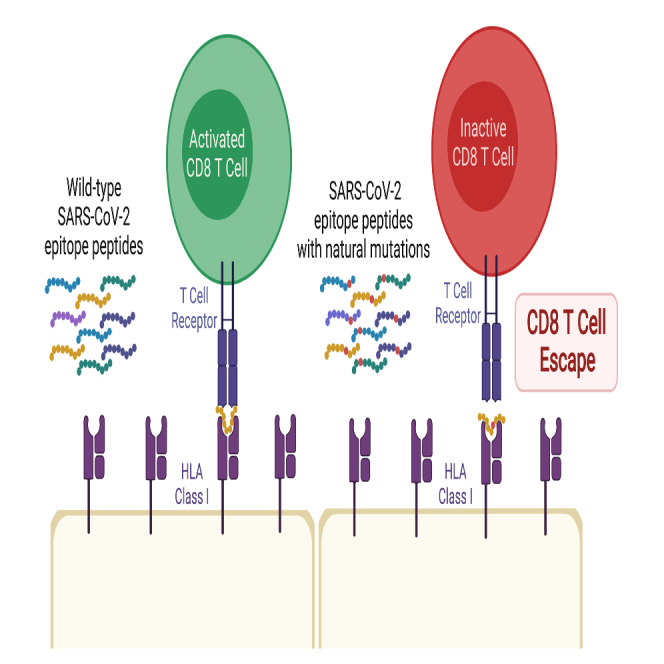

## Introduction

Evolution of SARS-CoV-2 can lead to evasion from adaptive immunity generated following infection and vaccination. Much focus has been on humoral immunity and spike protein mutations that impair the effectiveness of neutralizing monoclonal antibodies and polyclonal sera. T cells specific to conserved proteins play a significant protective role in respiratory viral infections such as influenza, particularly in broad heterosubtypic immunity (Hayward et al., 2015). T cell responses following SARS-CoV-2 infection are directed against targets across the genome and may play a role in favorable outcomes during acute infection and in immunosuppressed hosts with deficient B cell immunity (Huang et al., 2021; Peng et al., 2020; Tan et al., 2021). Although CD8+ T cells may not provide sterilizing immunity, they can protect against severe disease and limit risk of transmission, with a potentially more important role in the setting of antibody escape.

Little is known about the potential for SARS-CoV-2 mutations to impact T cell recognition. Escape from antigen-specific CD8+ T cells has been studied extensively in HIV-1 infection, where rapid intra-host evolution renders T cell responses ineffective within weeks of acute infection (Goonetilleke et al., 2009). Although these escape variants play an important role in the dynamics of chronic viral infections, the opportunities for T cell escape in acute respiratory viral infections are fewer and consequences are different. Nevertheless, several cytotoxic T-lymphocyte (CTL) escape variants have been described in influenza, such as the R384G substitution in the HLA B∗08:01-restricted nucleoprotein_380-388_ and B∗27:05-restricted nucleoprotein_383-391_ epitopes (Voeten et al., 2000). Long-term adaptation of influenza A/H3N2 has been demonstrated, with the loss of one CTL epitope every 3 years since its emergence in 1968 (Woolthuis et al., 2016).

## Results and discussion

### Amino acid variants within experimentally proven SARS-CoV-2 T cell epitopes

To explore the potential for viral evasion from SARS-CoV-2-specific T cell responses, we conducted a proof-of-concept study, focusing initially on identifying common amino acid mutations within experimentally proven T cell epitopes and testing the functional implications in selected immunodominant epitopes that we and others have described previously. We conducted a literature review in PubMed and Scopus databases (November 29, 2020; Data S4) that identified 14 publications defining 360 experimentally proven CD4+ and CD8+ T cell epitopes (Chour et al., 2020; Ferretti et al., 2020; Gangaev et al., 2020; Habel et al., 2020; Kared et al., 2021; Keller et al., 2020; Le Bert et al., 2020; Nelde et al., 2021; Peng et al., 2020; Poran et al., 2020; Schulien et al., 2021; Sekine et al., 2020; Shomuradova et al., 2020; Snyder et al., 2020). Of these, 53 that were described in ≥1 publication were all CD8+ epitopes (Table S1) and distributed across the genome (n = 14 open reading frame [ORF]1a, n = 5 ORF1b, n = 18 S, n = 2 M, n = 8 N, n = 5 ORF3a, n = 1 ORF7a). In total, 12,503 amino acid substitutions or deletions were identified within the 360 T cell epitopes by searching the mutation datasets downloaded from CoV-GLUE (http://cov-glue.cvr.gla.ac.uk/#/home) on July 30, 2021 (Figure S1 and Table S2). A total of 1,370 amino acid variants were present within the 53 CD8+ T cell epitopes with responses described across multiple cohorts, with at least one variant in all epitopes (Figure S2 and Table S3).

### Functional impact of variants within immunodominant SARS-CoV-2 T cell epitopes

**Figure 1:**
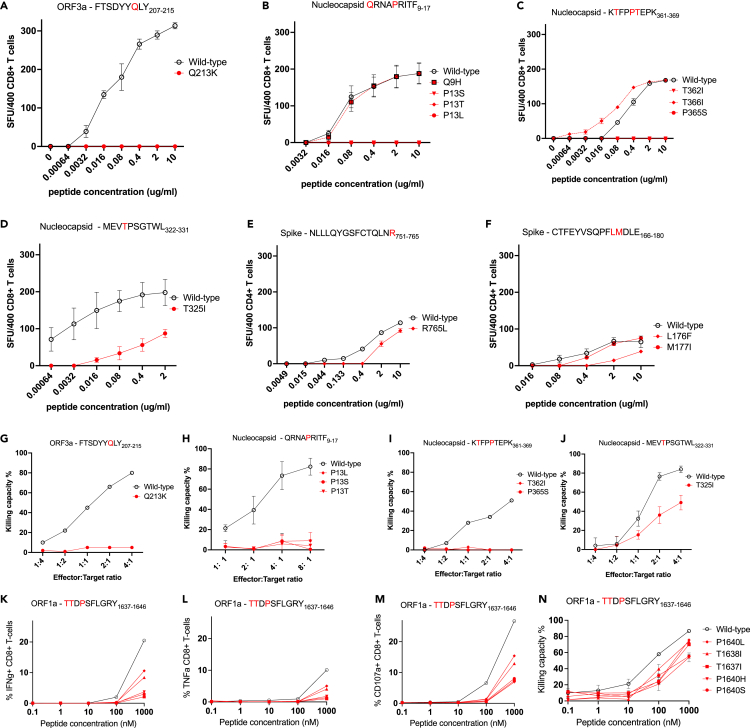
Functional impact of mutations in key SARS-CoV-2 dominant epitopes (A–F) Recognition of wild-type (black) and mutant (red) peptide titrations by polyclonal epitope-specific T cell lines in IFN-γ ELISpot assays. SFU, spot forming units. Shown are mean values from three or more replicates +/− standard deviation. (G–J) Ability of CD8+ T cell lines to kill autologous B cells loaded with wild-type (black) or mutant (red) peptides in carboxyfluorescein succinimidyl ester (CFSE) assays. The effector:target ratio denotes the proportion of CD8+ T cells:B cells in each assay. (K–N) Recognition of wild-type (black) and mutant (red) peptide titrations by a polyclonal CD8+ T cell line specific for the HLA∗A01:01-restricted ORF1a epitope TTDPSFLGRY_1637-1646_, using intra-cellular cytokine staining for interferon-gamma (IFNg, K), tumor necrosis factor (TNFa, L), and the degranulation factor CD107a (M), and a killing assay (N). Similar findings were seen with a T cell line generated from another donor (Figure S4).

We focused on evaluating the functional impact of variants within seven immunodominant epitopes in nucleocapsid, ORF3a, and spike (five CD8+, two CD4+) described in our study of UK convalescent donors (Peng et al., 2020), along with a further immunodominant ORF1a CD8+ epitope described in several other studies (Table 1). Of these, all six CD8+ epitopes have been described in at least two cohorts. In particular, responses to the A∗03:01/A∗11:01-restricted nucleocapsid KTFPPTEPK_361-369_ (Ferretti et al., 2020; Gangaev et al., 2020; Kared et al., 2021; Peng et al., 2020) epitope, A∗01:01-restricted ORF3a FTSDYYQLY_207-215_ (Ferretti et al., 2020; Kared et al., 2021; Peng et al., 2020; Schulien et al., 2021) epitope, and A∗01:01-restricted ORF1a TTDPSFLGRY_1637-1646_ (Ferretti et al., 2020; Gangaev et al., 2020; Nelde et al., 2021) epitope are consistently dominant and of high magnitude. We tested the functional avidity of SARS-CoV-2-specific CD4+ and CD8+ polyclonal T cell lines by interferon (IFN)-γ ELISpots using wild-type and variant peptide titrations (Figures 1A–1F). We found that several variants resulted in complete loss of responsiveness to the T cell lines evaluated: the Q213K variant in the A∗01:01-restricted CD8+ ORF3a epitope FTSDYYQLY_207-215_ (Ferretti et al., 2020; Kared et al., 2021; Peng et al., 2020; Schulien et al., 2021); the P13L, P13S, and P13T variants in the B∗27:05-restricted CD8+ nucleocapsid epitope QRNAPRITF_9-17_ (Nelde et al., 2021; Peng et al., 2020); and T362I and P365S variants in the A∗03:01/A∗11:01-restricted CD8+ nucleocapsid epitope KTFPPTEPK_361-369_ (Ferretti et al., 2020; Gangaev et al., 2020; Kared et al., 2021; Peng et al., 2020) (Figures 1A–1C).

**Table 1:** Epitopes and variants studied

Epitope	ORF	CD4/CD8	HLA	Variant	Frequency (%)	Countries	Global lineages	Loss of T cell response	References describing epitope
FTSDYYQLY_207-215_	3a	CD8	A∗01:01	Q213K	0.059	56	95	Yes	Ferretti et al. (2020); Kared et al., 2021; Peng et al. (2020); Schulien et al. (2021)^a^
QRNAPRITF_9-17_	N	CD8	B∗27:05	Q9H	0.289	74	176	No	Nelde et al. (2021); Peng et al. (2020)
QRNAPRITF_9-17_				P13L	0.978	97	194	Yes	Nelde et al. (2021); Peng et al. (2020)
QRNAPRITF_9-17_				P13S	0.210	83	132	Yes	Peng et al. (2020); Nelde et al. (2021)
QRNAPRITF_9-17_				P13T	0.102	45	81	Yes	Nelde et al. (2021); Peng et al. (2020)
MEVTPSGTWL_322-331_	N	CD8	B∗40:01	T325I	0.069	52	109	Partial	Nelde et al. (2021); Peng et al. (2020); Schulien et al. (2021)^b^
KTFPPTEPK_361-369_	N	CD8	A∗03:01 A∗11:01	T362I	0.293	85	165	Yes	Ferretti et al. (2020); Gangaev et al. (2020)^b^; Kared et al., 2021; Peng et al. (2020)
KTFPPTEPK_361-369_				T366I	0.237	74	154	No	Ferretti et al. (2020); Gangaev et al. (2020)^b^; Kared et al. (2021); Peng et al. (2020)
KTFPPTEPK_361-369_				P365S	0.794	73	142	Yes	Ferretti et al. (2020); Gangaev et al. (2020)^b^; Kared et al. (2021); Peng et al. (2020)
KCYGVSPTK_378-386_	S	CD8	A∗03:01	P384L	0.085	59	116	No	Ferretti et al. (2020); Peng et al. (2020)
CTFEYVSQPFLMDLE_166-180_	S	CD4	–	L176F	0.260	68	158	No	Peng et al. (2020)
CTFEYVSQPFLMDLE_166-180_				M177I	0.083	60	92	Partial	Peng et al. (2020)
NLLLQYGSFCTQLNR_751-765_	S	CD4	DRB1∗15:01	R765L	0.017	31	64	Partial	Peng et al. (2020)
					0.000				
TTDPSFLGRY1637-1646	ORF1A	CD8	A∗01:01	T1637I	0.189	58	113	Partial	Ferretti et al. (2020); Gangaev et al. (2020)^b^; Nelde et al. (2021)
TTDPSFLGRY1637-1646				T1638I	0.066	52	96	Partial	Ferretti et al. (2020); Gangaev et al. (2020)^b^; Nelde et al. (2021)
TTDPSFLGRY1637-1646				P1640S	0.202	69	165	Partial	Ferretti et al. (2020); Gangaev et al. (2020)^b^; Nelde et al. (2021)
TTDPSFLGRY1637-1646				P1640L	2.540	105	156	Partial	Ferretti et al. (2020); Gangaev et al. (2020)^b^; Nelde et al. (2021)
TTDPSFLGRY1637-1646				P1640H	0.050	27	20	Partial	Ferretti et al. (2020); Gangaev et al. (2020)^b^; Nelde et al. (2021)

Mutated positions detailed in red within wild-type epitope sequence. Frequency indicates % of sequences where variant is seen within the SARS-CoV-2 mutation dataset downloaded from CoV-GLUE (http://cov-glue.cvr.gla.ac.uk/#/home) on July 30, 2021. Global Lineages refers to Pango lineage assignment.

ORF, open reading frame; HLA, human leukocyte antigen.

a Responses to longer peptide also seen in Snyder et al. (2020).

b Responses to longer peptide also seen in Snyder et al. (2020) and Kared et al. (2021).

In contrast, Q9H in QRNAPRITF_9-17_, T366I in KTFPPTEPK_361-369_, P384L in the A∗03:01-restricted CD8+ spike epitope KCYGVSPTK_378-386_ (Ferretti et al., 2020; Peng et al., 2020), and M177I in the CD4+ spike epitope CTFEYVSQPFLMDLE_166-180_ (Peng et al., 2020) showed no impact on T cell recognition (Figures 1B, 1C, 1F, and S3). In fact, T366I in KTFPPTEPK_361-369_ appeared to result in higher avidity (Figure 1C). Several other variants showed partial loss of T cell responsiveness, with lower avidity observed to the variant peptide compared with wild-type peptide. These included T325I in the B∗40:01-restricted nucleocapsid epitope MEVTPSGTWL_322-331_ (Nelde et al., 2021; Peng et al., 2020; Schulien et al., 2021), R765L in the DRB1∗15:01-restricted CD4+ spike epitope NLLLQYGSFCTQLNR_751-765_ (Peng et al., 2020), and L176F in the CD4+ spike epitope CTFEYVSQPFLMDLE_166-180_ (Peng et al., 2020) (Figures 1D–1F). To confirm our findings, we evaluated the impact of CD8+ T cell epitope variants on CTL killing of peptide-loaded autologous B cells. Consistent with the ELISpot data, CTL killing ability was significantly impaired by Q213K in ORF3a FTSDYYQLY_207-215_; P13L, P13S, and P13T in nucleocapsid QRNAPRITF_9-17_; and T362I and P365S in nucleocapsid KTFPPTEPK_361-369_ (Figures 1G–1I). Partial impairment of killing ability was seen with T325I in MEVTPSGTWL_322-331_ (Figure 1J). Several variants within the dominant A∗01:01-restricted ORF1a epitope TTDPSFLGRY_1637-1646_ (Ferretti et al., 2020; Gangaev et al., 2020; Nelde et al., 2021) also resulted in partial loss of T cell recognition in both intra-cellular cytokine staining-based avidity assays (Figures 1K, 1L, 1M, and S4) and a killing assay (Figures 1N and S4).

### Potential mechanisms of loss of T cell recognition

T cell escape can occur via interrupting several mechanisms: antigen processing, binding of major histocompatibility complex (MHC) to peptide, or T cell receptor (TCR) recognition of the MHC-peptide complex. Although we did not explicitly establish which of these was responsible in each case, it is likely that any partial impairment of T cell recognition is due to reduced TCR binding to MHC-peptide. Reasons for complete escape are more difficult to predict. As the anchor residues of peptide-MHC binding in A∗03:01/A∗11:01-restricted KTFPPTEPK_361-369_ are at positions 2 and 9, T362I (position 2) may impair peptide-MHC binding, while P365S (position 5) may affect a T cell binding residue (Rammensee et al., 1999). The proline changes (P13L, P13S, P13T) in the B∗27:05-restricted QRNAPRITF_9-17_ (position 5) again may be at a key T cell contact residue, as peptide-MHC binding anchor residues are at positions 2 and 9 (Rammensee et al., 1999). The anchor residues for the A∗01:01-restricted FTSDYYQLY_207-215_ are predicted to be at positions 3 and 9, with auxiliary anchors at positions 2 and 7 (Rammensee et al., 1999), which may explain the impact of the Q213K (position 7) variant. In keeping with this, we see no significant impact of these mutations on the predicted binding affinities of epitope to MHC (Table S4). Despite a modest 4-fold decrease in predicted IC_50_ for Q213K compared with wild type, FTSDYYKLY_207-215_ is still a strong binder to A∗01:01.

*Ex vivo* IFN-γ ELISpots in two A∗03:01 and two B∗27:05 convalescent donors confirmed loss of responses to variant peptides seen with T cell lines specific to KTFPPTEPK_361-369_ and QRNAPRITF_9-17_ (Figure S5). Thus, our findings using T cell lines are representative of the circulating T cell response to these epitopes and of physiological relevance. Of interest, one A∗03:01 donor had low-level responses to P365S and T362I, suggesting that subdominant responses via alternative TCR are possible.

### T cell receptor diversity in CD8+ T cell lines with loss of epitope recognition due to amino acid variants

**Figure 2:**
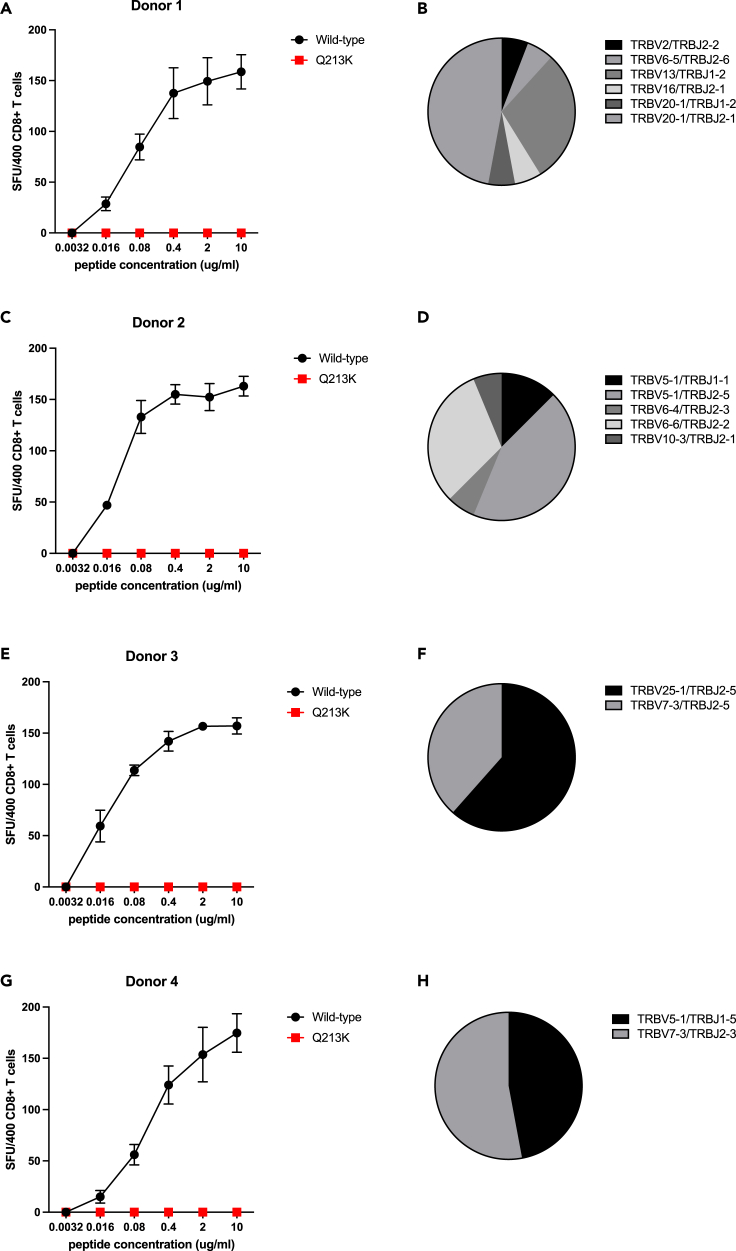
T cell receptor (TCR) repertoire of polyclonal CD8+ T cell lines specific for A∗01:01-restricted ORF3a epitope FTSDYYKLY_207-215_ (A, C, E, and G) Data are shown from T cell lines generated using peripheral blood mononuclear cells from four donors. The Q213K variant (red) showed complete loss of recognition in each case using peptide titrations in IFN-γ ELISpot assays compared with recognition of the wild-type (black) peptide. Shown are mean values from three or more replicates +/− standard deviation. SFU, spot forming units. (B, D, F, and H) A diverse range of TCRs was observed.

TCR sequencing of polyclonal CD8+ T cell lines specific for the FTSDYYKLY_207-215_ epitope and B∗27:05-restricted QRNAPRITF_9-17_ epitope was undertaken to explore whether the complete loss of T cell recognition observed was dependent on specific TCRs. A diverse range of TCRs was found in FTSDYYKLY_207-215_ T cell lines from four donors (Figure 2), demonstrating that Q213K results in escape from several TCRs. Similar findings were seen in TCR data from a B∗27:05-restricted QRNAPRITF_9-17_ T cell line from one donor (Figure S6). It is worth noting that our data are biased by using T cell lines generated from donors recruited early in the pandemic and therefore likely infected with “wild-type” viruses (i.e., lineage B or B.1 viruses) (Peng et al., 2020). Although variants that impair antigen processing or MHC-peptide binding result in irreversible loss of T cell recognition, CTLs with new TCR repertoires can overcome TCR-mediated escape variants, as has been described in HIV-1 infection (Ladell et al., 2013).

### Frequency of epitope variants over time and appearance in global SARS-CoV-2 phylogeny

**Figure 3:**
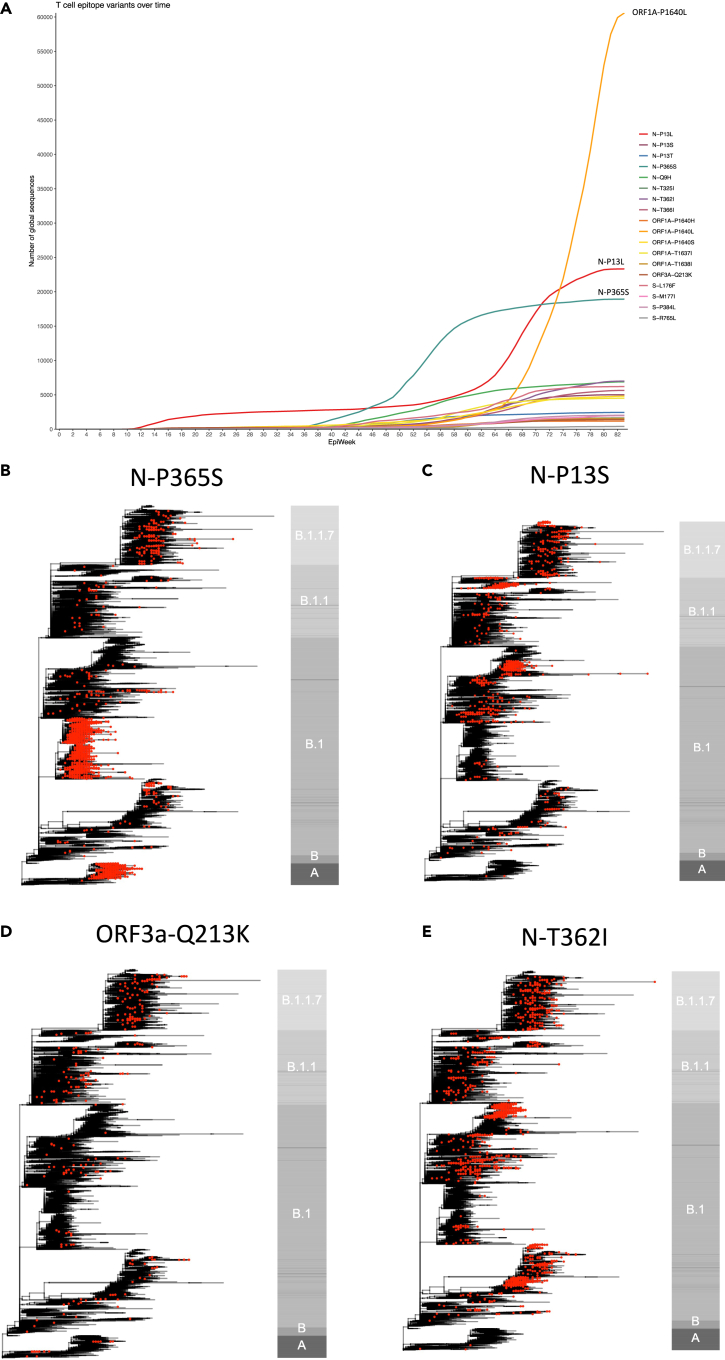
Global presence of variants in key dominant SARS-CoV-2 epitopes (A) Weekly frequency over time since beginning of SARS-CoV-2 pandemic of all variants studied in functional experiments. Mutation counts were obtained from COG-UK global metadata (dated August 4, 2021). Variants named with prefix of SARS-CoV-2 protein (S, spike; N, nucleocapsid), followed by wild-type amino acid, position within protein, and variant amino acid. Epiweek relates to week number since start of global SARS-CoV-2 pandemic was declared on March 11, 2020. (B–E) Phylogenies representing global SARS-CoV-2 genomes depicting the presence of epitopes variants impacting T cell responses. In each case, phylogenies represent all available variant sequences (red tips), along with a selection of non-variant sequences, which were subsampled for visualization purposes. The bar to the right of each phylogeny is annotated by main ancestral lineages only and not each individual PANGO lineage that viruses belong to. The grapevine pipeline (https://github.com/COG-UK/grapevine) was used for generating the phylogeny based on all data available on GISAID and COG-UK up until August 4, 2021.

Many variants examined in our study were at relatively low frequency and stable prevalence at the time of writing, other than P365S in KTFPPTEPK_361-369,_ P1640L in TTDPSFLGRY_1637-1646_, and variants affecting the proline at position 13 in QRNAPRITF_9-17_ (Table 1 and Figure 3A). We explored whether variants that result in loss of T cell recognition appeared as homoplasies in the phylogeny of SARS-CoV-2 suggestive of repeated independent selection, or whether global frequency is due mainly to the expansion of lineages after initial acquisition. Although in some cases variant frequency was dependent on a few successful lineages, P365S, Q213K, T362I, P13L, P13S, and P13T had arisen independently on several occasions including within the B.1.1.7 lineage (Figures 3B, 3C, 3D, 3E, S7A, and S7B). It is important to emphasize that this homoplasy and our functional data do not prove selection due to T cell escape, which would require demonstration of intra-host evolution. The positions we find important for T cell recognition may be under selective pressure for reasons other than T cell immunity. A recent study has documented intra-host evolution of minority variants within A∗02:01 and B∗40:01 CD8+ epitopes that impair T cell recognition, although not all epitopes are dominant and very few of the variants studied were represented among the global circulating viruses (Agerer et al., 2021).

### Conclusions

There is unlikely to be adequate population immunity at present to see global changes due to T cell selection akin to what has been seen in adaptation of H3N2 influenza over time (Woolthuis et al., 2016). Furthermore, polymorphism in HLA genes restricts the selective advantage of escape within one particular epitope to a relatively small proportion of the population, given the breadth in T cell responses we and others have shown. The polyclonal T cell response in a given individual is therefore unlikely to be diminished significantly by mutations present in any one circulating variant, unlike the potential impact on neutralizing antibody responses seen with mutations in the spike protein. Nevertheless, responses to many of the CTL epitopes we have studied are dominant within HLA-matched individuals across many cohorts (Peng et al., 2020). As A∗03:01, A∗11:01, and A∗01:01 are common HLA alleles globally, loss of T cell responses to dominant epitopes such as KTFPPTEPK_361-369_ and FTSDYYQLY_207-215_ may be significant. Substitution of three different amino acid variants at nucleocapsid position 13 within the B∗27:05-restricted QRNAPRITF_9-17_ epitope is also striking and suggests significant positive selective pressure at this site. Successful maintenance of these substitutions within some lineages also suggest that this is a position where such amino acid changes are tolerated with limited impact on the virus life cycle. A single dominant, protective B∗27:05-restricted epitope has been described in HIV-1 infection, with T cell escape associated with progression to AIDS. T cell escape from a B∗27:05-restricted influenza A epitope (nucleoprotein_383-391_) has also been observed (Voeten et al., 2000).

A significant increase in sites under diversifying positive selective pressure was observed around November 2020, most notably in ORF3a, N, and S (Martin et al., 2021). As vaccine and naturally acquired population immunity increase further, the frequency of variants we have described should be monitored globally, as well as further changes arising within all immunodominant T cell epitopes. We have recently incorporated the ability to identify spike T cell epitope variants in real-time sequence data into the COG-UK mutation explorer dashboard (http://sars2.cvr.gla.ac.uk/cog-uk/). Non-spike T cell immune responses will also become increasingly important to vaccine-induced immunity as inactivated whole-virus vaccines are rolled out. Our findings demonstrate the potential for T cell evasion and highlight the need for ongoing surveillance for variants capable of escaping T cell as well as humoral immunity.

### Limitations of the study

We have chosen to focus on key SARS-CoV-2 immunodominant epitopes characterized early in the pandemic, and further epitopes have been identified since. It would be important to assess mutations of increasing prevalence within all immunodominant epitopes in the future to provide a comprehensive overview of potential SARS-CoV-2 T cell escape. Although our findings suggest that reduced T cell receptor binding to MHC-epitope complex is likely responsible for the most striking impact of mutations on T cell responses we describe, this needs to be demonstrated experimentally. Finally, further studies are required to demonstrate the occurrence of T cell escape within individuals and establish how frequently this occurs. Given the potential for immune escape in prolonged or chronic SARS-CoV-2 infections that could give rise to new variants of concern, a focus on infections in immunocompromised individuals would be important.

## STAR★Methods

### Key resources table

**Table:** 

REAGENT or RESOURCE	SOURCE	IDENTIFIER
**Antibodies**		

PE-Cy7 anti-human CD107a (H4A3)	BD Biosciences, UK	Cat#561348; RRID:AB_10644018
BV510 anti-human CD3 (UCHT1)	BD Biosciences, UK	Cat#563109; RRID:AB_2732053
BV421 anti-human CD8 (RPA-T8)	BD Biosciences, UK	Cat#562428; RRID:AB_11154035
PE anti-human TNFa (MAb11)	Thermo Fisher Scientific	Cat#12-7349-82; RRID:AB_466208
FITC anti-human IFNγ (45-15)	Miltenyi Biotec Ltd	Cat# 130-091-641; RRID:AB_244194
FITC anti-human CD4 (SK3&SK4)	BD Bioscience, UK	Cat#347413; RRID:AB_400297
FITC anti-human CD8 (SK1)	BD Bioscience, UK	Cat#347313; RRID:AB_400279
BV421 anti-human CD19 (HIB19)	Biolegend, UK	Cat#302234; RRID: AB_10897802

**Chemicals, peptides, and recombinant proteins**		

Synthesized peptides	GenScript Biotech, Netherlands	NA
QRNAPRITF-B∗27:05 pentamer	Proimmune	Cat#4354
KTFPPTEPK-A∗03:01 pentamer	Proimmune	Cat#4356A
MEVTPSGTWL-B∗40:01 pentamer	Proimmune	Cat#4328
FTSDYYQLY-A∗0101 pentamer	Proimmune	Cat#4355
KTFPPTEPK-A∗11:01 pentamer	Proimmune	Cat#4356B
NLLLQYGSFCTQLNR-DRA∗01:01/DRB1∗15:01 tetramer	Proimmune	NA (Custom)
IL-2/TCGF	Helvetica healthcare	Cat#0801017
IL-7	Biotechne, UK	Cat#207-IL

**Critical commercial assays**		

Human IFN-γ ELISpot BASIC kit	Mabtech	Cat#3420-2A
SMARTer® RACE 5’/3′ kit	TaKaRa	Cat#634858
Monarch DNA gel extraction kit	New England BioLabs	Cat#T1020S
TOPO™ TA Cloning™ kit for sequencing	Thermo Fisher Scientific	Cat#K457501
RNeasy plus mini kit	Qiagen	Cat#74134
Advantage2 PCR kit	TaKaRa	Cat#639207
LIVE/DEAD fixable near-IR dead cell stain kit	Thermo Fisher Scientific	Cat#L34975
CellTrace™ CFSE cell proliferation kit	Thermo Fisher Scientific	Cat#C34554
CellTrace™ violet cell proliferation kit	Thermo Fisher Scientific	Cat#C34557
eBioscience™ 7-AAD viability staining solution	Thermo Fisher Scientific	Cat#00-6993-50
QIAprep spin miniprep kit	Qiagen	Cat#27106

**Deposited data**		

GLUE mutation dataset – replacement (In supplementary information Mutation_identification, input_data)	CoV-GLUE	http://cov-glue.cvr.gla.ac.uk/#/home
GLUE mutation dataset – deletion (In supplementary information Mutation_identification, input_data)	CoV-GLUE	http://cov-glue.cvr.gla.ac.uk/#/home
COG-UK metadata (original data is unpublished and restricted by the data sharing agreement with COG-UK, data provided in supplementary information Tree_visualisation (input_data) has been cleaned for the purpose of public sharing and includes all publicly available variables required to recreate the analysis described)	COG-UK	https://www.cogconsortium.uk/tools-analysis/public-data-analysis-2/
Code for identifying mutation (In supplementary information Mutation_identification)	Custom	NA
Code for plotting variant prevalence (In supplementary information Variant_prevalence)	Custom	NA
Code for plotting global phylogenetic tree (In supplementary information Tree_visualisation)	Custom	NA

**Experimental models: Cell lines**		

Lymphoblastoid cell lines (killing assay target cells) – transformed with epstein-Barr virus and ciclosporin A from healthy donors. Cell lines like this are a standard reagent in many laboratories. Cells are available upon request, contingent upon material transfer agreement and necessary ethical permissions.	Custom	NA

**Oligonucleotides**		

Primer 5′-TGCTTCTGATGGCTCAAACAC AGCGACCT-3′	Custom	NA

**Software and algorithms**		

Flowjo 10.7.1	BD Biosciences	NA
NetMHCpan 4.1	Reynisson et al. (2020)	http://www.cbs.dtu.dk/services/NetMHCpan/
R (version 3.5.3)	R Core Team, 2021	https://www.r-project.org
GraphPad prism 9	GraphPad	NA
Biorender	Science Suite inc	www.biorender.com

**Other**		

FacsCanto II cytometer	BD Biosciences UK	NA

### Resource availability

#### Lead contact

Further information and requests for resources and reagents should be directed to and will be fulfilled by the lead contact, Thushan de Silva (t.desilva@sheffield.ac.uk).

#### Materials availability

This study did not generate new unique reagents.

### Experimental model and participant details

#### Participants

SARS-CoV-2 recovered donors were recruited in Oxford into the Sepsis Immunomics study (Ref 13/SC/0296) and in Liverpool into the ISARIC study (Ref 13/SC/0149). Both studies were granted ethical approval from the South Central - Oxford C Research Ethics Committee in England. The age and sex of donors used in this study are detailed below.

**Table:** 

Participant	Sex	Age
Donor1	M	73
Donor2	F	45
Donor3	M	56
Donor4	F	57
Donor5	M	69
Donor6	M	53
Donor7	M	61
Donor8	M	50
Donor9	F	44
Donor 10	M	46
Donor 11	F	58

#### Isolation of peripheral blood mononuclear cells

Blood from participants was collected in EDTA anticoagulant tubes, layered onto an equal volume of lymphoprep (Stemcell) in a falcon tube at room temperature, then centrifuged at 800 × g for 20 min at room temperature with the brake off. Peripheral Blood Mononuclear Cells (PBMCs) were aspirated at the plasma:lymphoprep interface and washed twice with RPMI medium.

Isolated PBMCs were cultured in RPMI (GIBCO) with 10% (v/v) fetal bovine serum (FBS), 100 units/mL penicillin, 0.1 mg/mL streptomycin at 37°C in 5% carbon dioxide (CO_2_) and used to generate polyclonal T cell lines and lymphoblastoid cell lines.

#### Generation of polyclonal T cell lines

1–2 million PBMCs were seeded per well in a 24-well plate in RPMI (GIBCO) with 10% (v/v) human serum, 10% (v/v) IL-2/TCGF (Helvetica healthcare), 5 ng/mL IL-7 (Biotechne). Peptides were added at 10 ug/mL. Cells were fed on day 4 or earlier if media the turned yellow and then every 4 days. On day 14, antigen-specific CD8+ T-cells were sorted with pentamer staining and CD4+ T-cells were sorted using tetramer staining. Subsequently, sorted cells were plated in a 96-well U-bottom plate with 100–1000 cells/well and fed with 200,000 irradiated allogeneic PBMCs with 50ug/mL phytohemagglutinin (PHA).

#### Generation of lymphoblastoid cell lines

2–2.5 million PBMC were resuspended in 1 mL supernatant from B95-8 cells and added to 24 well plate, then incubated 4–5 h at 37°C in 5% CO_2_. Following incubation 1 mL of RPMI (GIBCO) with 20% (v/v) FBS, 100 units/mL penicillin, 0.1 mg/mL streptomycin was added to each well and ciclosporin A (CSA) added to a final concentration of 100 ng/mL. Cells were fed every 4–6 days and lines expanded when required.

#### Identification of amino acid variants within T cell epitopes

Variants within the 360 experimentally proven T cell epitopes were identified using mutation datasets downloaded from CoV-GLUE (http://cov-glue.cvr.gla.ac.uk/#/home) on the 30^th^ July 2021. Both amino acid substitutions and deletions were considered in this study. Sequences were excluded if they did not contain a start and/stop codon at the beginning and end of each ORF. COG-UK global metadata downloaded on 04^th^ August 2021 was used to plot the variant over time (Figure 2A). Sequence positions mentioned in this study are relative to Wuhan-Hu-1 (GenBank accession MN908947.3) and were compared using custom R scripts (R version 3.5.3).

#### Peptide titrations using T cell lines and IFN-γ ELISpot assays

Polyclonal CD4+ and CD8+ T cell lines specific for seven previously described immunodominant epitopes (Peng et al., 2020) were generated after MHC class I Pentamer or MHC class II tetramer sorting from cultured short-term cultures of SARS-CoV-2 recovered donor PBMCs. Antigen-specific T-cells were confirmed by corresponding Pentamer or tetramer staining. T-cells were stained with Live/Dead dye (Thermo Fisher Scientific, UK), then stained with pentamer or tetramer, followed by CD8-FITC (BD Bioscience, UK) or CD4-FITC (BD Bioscience, UK) staining. The functional avidity of T cell lines was assessed by IFN-γ ELISpot assays (Peng et al., 2015). T cell lines were stimulated with wild-type and variant peptide-pulsed autologous B-cells, starting at 10 μg/mL and serial 1:5 dilutions using 400 cells T cells and 20,000 B cells per condition at 37°C for 6 h. Peptides were synthesised by GenScript Biotech (Netherlands) B.V. To quantify antigen-specific responses, spots of the control wells, containing no peptide, were subtracted from test wells and results expressed as spot forming units (SFU) per 400 T-cells. If negative control wells had >30 SFU/T-cells or positive control (PHA) were negative, results were considered invalid. Duplicate wells were used for each test and results are from three to seven independent experiments.

#### *Ex vivo* IFN-γ ELISpots in SARS-CoV-2 recovered donors

Cryopreserved PBMCs were used from SARS-CoV-2 recovered donors for *ex vivo* IFN-γ ELISpots with wild-type and variant peptides. Peptides were added to 200,000 PBMCs at a final concentration of 2 μg/mL for 16–18 h (two replicates per condition). Results were interpreted as detailed above. PBMCs used were from samples taken when patients were between 35 and 53 days from symptom onset.

#### Peptide titrations using T cell lines and intra-cellular cytokine staining

The functional avidity of polyclonal CD8+ T cell lines specific for the ORF1a epitope TTDPSFLGRY_1637-1646_ (Ferretti et al., 2020; Gangaev et al., 2020; Nelde et al., 2021) was assessed using stimulation with wild-type and variant peptides starting at 1000 nM and serial 1:10 dilutions, followed by intra-cellular cytokine staining (ICS). 1–1.5 × 10^6^ cells were plated in R10 in a 96 well U-bottom plate and peptide added. DMSO was used as the negative control at the equivalent concentration to the peptides. Degranulation of T cells (a functional marker of cytotoxicity) was measured by the addition of an anti-CD107a-PE-Cy7 antibody (clone H4A3, BD Biosciences, UK) at 1 in 20 dilution during the culture. The cells were then incubated at 37°C, 5% CO_2_ for 1 h before adding Brefeldin A (10 μg/mL). Samples were incubated at 37°C, 5% CO_2_ for a further 5 h before proceeding with staining for flow cytometry. Cells were stained with a cell viability dye (near infrared, Thermo Fisher Scientific, UK) at 1:500 then fixed in 2% formaldehyde for 20 min, followed by permeabilization with 1× Perm/Wash buffer (BD Biosciences). Staining was performed with the following antibodies: anti-CD3-BV510 (clone UCHT1, BD Biosciences), anti-CD8-BV421 (clone RPA-T8, BD), TNF-PE (clone MAb11, Thermo Fisher Scientific) and anti-IFN-γ-FITC (clone 45-15, Miltenyi Biotec Ltd, UK). Samples were run on a FacsCanto II cytometer and the data were analyzed using FlowJo software version 10 (BD Biosciences). During analysis, exclusion of doublet cells was performed, followed by gating on live peripheral blood mononuclear cells and estimation of the % of CD3+CD8+ T-cells expressing cytokines at each peptide concentration.

#### Cytotoxic T-lymphocyte (CTL) killing assays

Killing assays were performed in one of two ways. (1) For T cell lines characterised using IFN-γ ELISpot assays, autologous B-cells were stained with 0.5 μmol/L carboxyfluoroscein succinimidyl ester (CFSE, Thermo Fisher Scientific) before wild-type or variant peptide loading at 1 μg/mL for one hour. Peptide-loaded B-cells were co-cultured with CTLs at a range of effector:target (E:T) ratios from 1:4 to 8:1 at 37°C for 6 h and cells stained with 7-AAD (eBioscience, UK) and CD19-BV421 (clone HIB19, Biolegend, UK). Assessment of cell death in each condition was based on the CFSE/7-AAD population present. (2) For the ORF1a epitope TTDPSFLGRY_1637-1646_ (Ferretti et al., 2020; Gangaev et al., 2020; Nelde et al., 2021) characterised using ICS, appropriately HLA-matched peptide-loaded B-cells were used as target cells and labeled with CFSE according to the manufacturer's protocol. Briefly, cells were pulsed with varying concentrations of peptide (1000 nM followed by 1:10 serial dilutions) for one hour at 37°C. Unpulsed cells were labeled with Cell Trace violet (CTV; Molecular Probes). Pulsed and unpulsed cells were mixed in a 1:1 ratio and 50 ul added to 50 ul of peptide-specific short-term T cell lines and incubated in a 96 well plate for 10 h at an effector/target ratio of 10:1 in duplicates. After incubation, cells were stained with near IR viability marker, CD3 (clone UCHT1; eBioscience), CD8 (clone RPA-T8; eBioscience), and CD19 (clone LT19; Mitenyi Biotec). The mean percent survival of CFSE-labelled cells in wells containing no effector cells was used to calculate the expected frequency of target cells in each well: expected ratio (ER) was calculated as %CFSE^+^/%CTV^+^. The specific killing was then calculated as: % Specific killing = 11 × [(ER x %CTV^+^ cells) - %CFSE + cells]/(ER x %CTV^+^ cells).

#### Predictions of binding strength of peptides to MHC

NetMHCpan 4.1 (http://www.cbs.dtu.dk/services/NetMHCpan/, Reynisson et al., 2020) was used to predict the binding strength of wild type and variant epitopes under standard settings (strong binder % rank 0.5, weak binder % rank 2). The predicted affinity (IC_50_ nM) for variant epitopes was compared with wild type.

#### T cell receptor (TCR) sequencing

One million cells from each epitope-specific polyclonal CD8+ T-cell line were harvested and washed three times with Phosphate Buffered Saline. Total RNA was extracted using the RNeasy Plus Mini kit (Qiagen, Germany), and cDNA was then synthesized from 300 ng RNA using the SMARTer RACE cDNA amplification kit (Takara Bio, Japan) following the manufacturer's instruction. Subsequently, cDNA was amplified for variable regions of the TCR-β chain using the PCR Advantage kit (Takara Bio), with the primer 5′-TGCTTCTGATGGCTCAAACACAGCGACCT-3′ and run on a 1.2% agarose gel for PCR band confirmation (at 500 bp). PCR products were purified using the Monarch DNA Gel Extraction kit (New England BioLabs, USA) and then transformed into TOP10 competent cells (ThermoFisher). Plasmid DNA was extract using the Spin Miniprep kit (Qiagen) followed by Sanger sequencing.

#### Phylogenetic tree generation

Phylogenies were generated using the grapevine pipeline (https://github.com/COG-UK/grapevine) based on all data available on GISAID and COG-UK up until 8^th^ August 2021. To visualise all sequences with a specific amino acid variant of interest in a global context, a representative sample of global sequences was obtained in two steps. First, one sequence per country per epi week was selected randomly, followed by random sampling of the remaining sequences to generate a sample of 6000 down-sampled sequences. The global tree was then pruned using code adapted from the tree-manip package (https://github.com/josephhughes/tree-manip).

The tips of sequences with amino acid variants impacting T cell recognition were colour-coded. Visualisations were produced using R/ape, R/ggplot2, R/ggtree, R/treeio, R/phangorn, R/stringr, R/dplyr, R/aplot.

### Quantification and statistical analysis

Mean and standard deviation for replicates used in T cell experiments were calculated and plotted using GraphPad Prism version 9. No quantitative statistical analysis was undertaken in this manuscript.

## Data Availability

• Additional Supplemental Items are available from Mendeley Data at https://data.mendeley.com/datasets/8gyvpj4wsc/draft?a=b1ce80de-e208-443e-a839-6b1852dafb63 • Code and data used for identifying mutations within T cell epitopes are provided in Data S1 Mutation identification, related to all figures. The analysis folder contains an R code used for data manipulation and two sub-folders: *input_data* and *output.* Mutation datasets downloaded from CoV-GLUE are provided in the *input_data* sub-folder. • Code and data used for plotting the variant prevalence over time are provided in Data S2 Variant prevalence, related to Figure 3. The analysis folder contains an R code and two sub-folders: *input_data* and *output.* Mutation counts obtained from COG-UK global metadata are provided in the *input_data* folder. • Code and data used for plotting the global phylogenies representation are provided in Data S3 Tree visualisation, related to Figure 3. The analysis folder contains an R code and two sub-folders: *input_data* and *output.* COG-UK metadata and lists of sequences with our mutations of interest are provided in the *input_data* sub-folder. • Any additional information required to re-analyze the data reported in this paper is available from the lead contact upon request. The graphical abstract was created with Biorender.

## Supplemental information

Document S1. Figure S1–S7 and Table S4

Table S1. 360 experimentally proven T-cell epitopes identified during the literature review, along with CD4+ vs CD8+ epitope status and HLA restriction where known

Table S2. 12503 amino acid variants identified within the 360 experimentally proven T cell epitopes

Table S3. Amino acid variants identified within the 53 experimentally proven CD8+ T cell epitopes described in >1 cohort, along with all 53 experimentally proven CD4+ T cell epitopes

Data S1. Mutation_identification R Code and input data used for identifying mutations within T cell epitopes

Data S2. Variant_prevalence R code and input data used for plotting the prevalence of variants within T cell epitopes over time

Data S3. Tree_visualization R code and putput data used for plotting representative global phylogenies, highlighting the presence of the variant of interest
