# Supplementary material for: The impact of viral mutations on recognition by SARS-CoV-2 specific T cells
Source: iScience. 2021 Oct 28;24(11):103353. doi: 10.1016/j.isci.2021.103353 (PMC8552693; doi:10.1016/j.isci.2021.103353)
Supplement: Document S1. Figure S1–S7 and Table S4 [file mmc1.pdf]

iScience, Volume *24*

Supplemental information

The impact of viral mutations on recognition by SARS-CoV-2 specific T cells

Thushan I. de Silva, Guihai Liu, Benjamin B. Lindsey, Danning Dong, Shona C. Moore, Nienyun Sharon Hsu, Dhruv Shah, Dannielle Wellington, Alexander J. Mentzer, Adrienn Angyal, Rebecca Brown, Matthew D. Parker, Zixi Ying, Xuan Yao, Lance Turtle, Susanna Dunachie, COVID-19 Genomics UK (COG-UK) Consortium, Mala K. Maini, Graham Ogg, Julian C. Knight, ISARIC4C Investigators, Yanchun Peng, Sarah L. Rowland-Jones, and Tao Dong

Supplementary information

Data S4. Search terms used for literature review of SARS-CoV-2 T-cell epitopes in PubMed and Scopus databases, related to Table 1.

(TITLE-ABS-KEY(("nCov" OR "Novel Coronavirus" OR "2019 Novel Coronavirus" OR "Covid-19" OR "2019-nCoV" OR "Severe Acute Respiratory Syndrome- Coronavirus-2" OR "SARS-CoV-2")) AND TITLE-ABS-KEY(("T-cell*" OR "Tcell*" OR "T Cell*" OR "Peptide" OR "cd4+" OR "cd8+" OR "CD8+" OR "CD4+" OR "t-lymphocyt*" OR "T Lymphocyte*")) AND TITLE-ABS-KEY("Epitope*"))

<u>Reasons for rejection of 271/285 publications identified</u>

1. Did not investigate SARS-CoV-2 T-cell responses on convalescent donors (included B-cell and antibody responses, responses to other viruses e.g. SARS, MERS). N = 53.
2. SARS-CoV-2 T-cell responses explored with whole protein and/or overlapping peptides, with no individual epitope-specific data. N = 31.
3. SARS-CoV-2 T-cell epitopes described soley with bioinformatic prediction (not experimentally proven). N = 129.
4. Reviews of SARS-CoV-2 T-cell responses. N = 44.
5. SARS-CoV-2 T-cell responses in non-human studies. N = 6.
6. Other. N = 7.

Of note, while some publications had defined optimal T-cell epitopes and HLA restriction, some simply reported the sequence of the longer overlapping peptides containing potential epitope to which responses were seen.

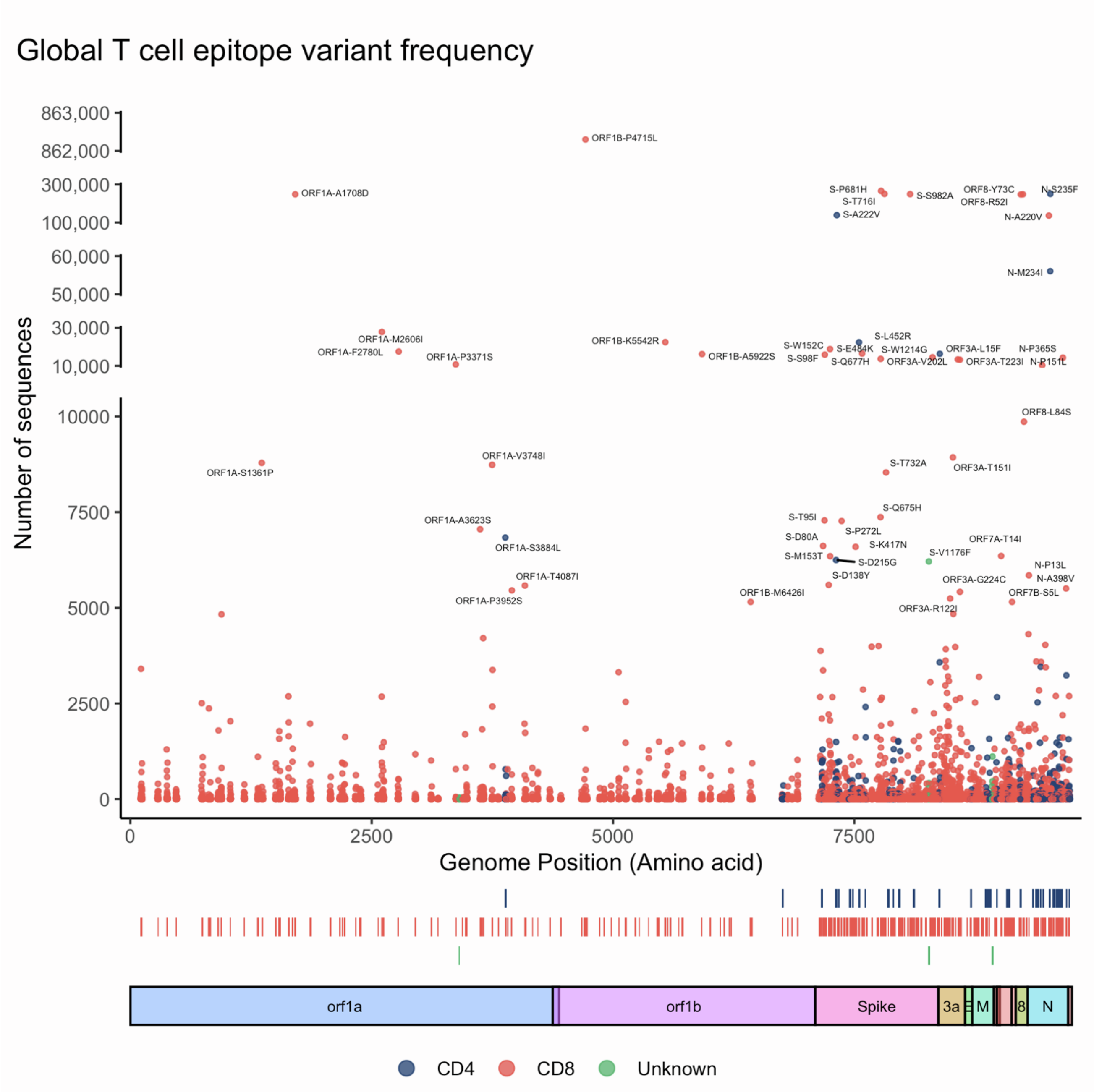

Figure S1. Frequency of amino acid variants within experimentally proven T-cell epitopes identified in the literature, related to Table 1 and Figure 3. Variants within 360 epitopes identified by searching the mutation datasets downloaded from CoV-GLUE on the 30th July 2021. CD4 and CD8 epitopes are coloured in blue and red respectively. Open reading frame (ORF) and variant annotated for mutations of higher frequency following convention of wild type amino acid, followed by amino acid position within each ORF and replacement amino acid (e.g. P681H for a proline to histidine at position 681).

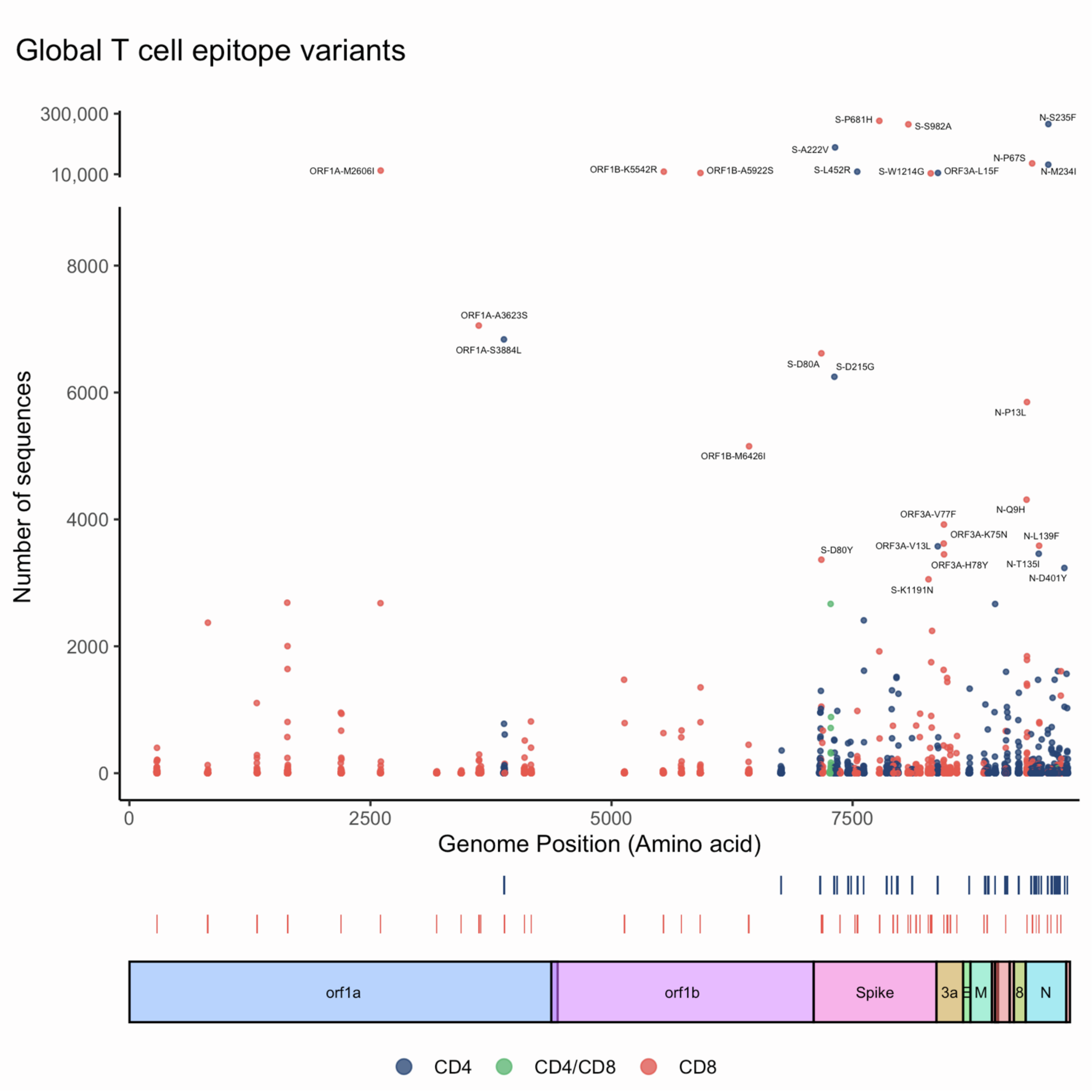

Figure S2. Frequency of amino acid variants within a focused set of experimentally proven T-cell epitopes identified in the literature, related to Table 1 and Figure 3. Variants in epitopes identified by searching the mutation datasets downloaded from CoV-GLUE on the 30th July 2021. Shown are variants within CD8+ T-cells epitopes described in two or more cohorts (n=53), as well as all experimentally proven CD4+ T-cell epitopes (n=53). CD4 and CD8 epitopes are coloured in blue and red respectively. Open reading frame (ORF) and variant annotated for mutations of higher frequency following convention of wild type amino acid, followed by amino acid position within each ORF and replacement amino acid (e.g. P681H for a proline to histidine at position 681).

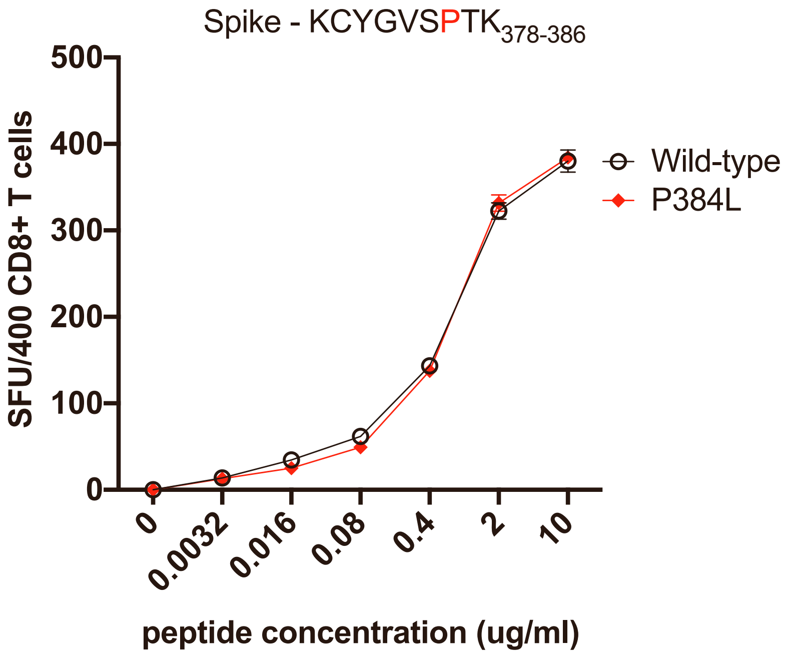

Figure S3. IFN-γ ELISpot responses to CD8+ T-cell line specific to the A*03:01-restricted spike epitope KCYGVSPTK$_{378\text{-}386}$ using wild-type and P384L mutant peptide titrations, related to Table 1 and Figure 1. n=3 replicates are used per condition. SFU = spot forming units. Mean and standard deviation displayed.

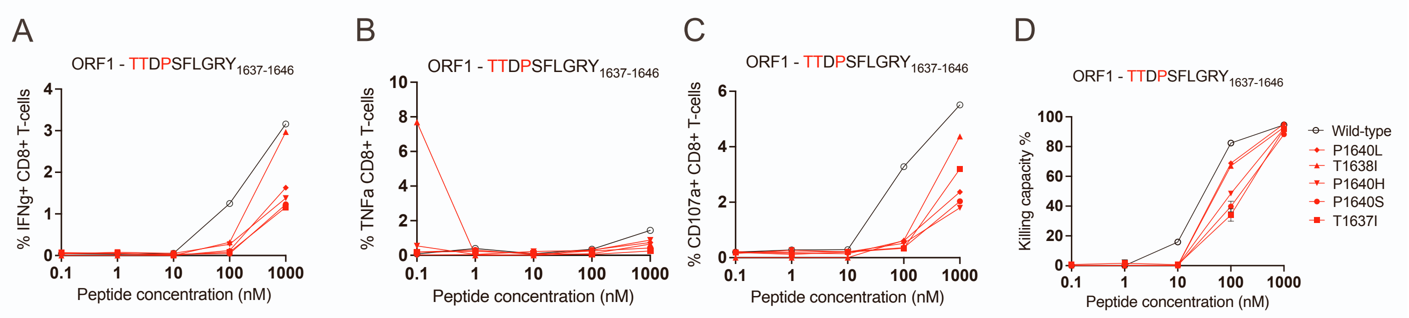

Figure S4. Recognition of wild-type (black) and mutant (red) peptide titrations by a polyclonal CD8+ T-cell line specific for the HLA*A01:01-restricted ORF1a epitope TTDPSFLGRY$_{1637\text{-}1646}$, related to Figure 1. Intra-cellular cytokine staining for interferon-gamma (IFNg, A), tumour necrosis factor (TNFa, B) and the degranulation factor CD107a (C) are shown to measure T-cell recognition, as well as a killing assay (D). Results confirmed those seen with a T-cell line generated from peripheral blood mononuclear cells from another donor, shown in Figure 1K – N.

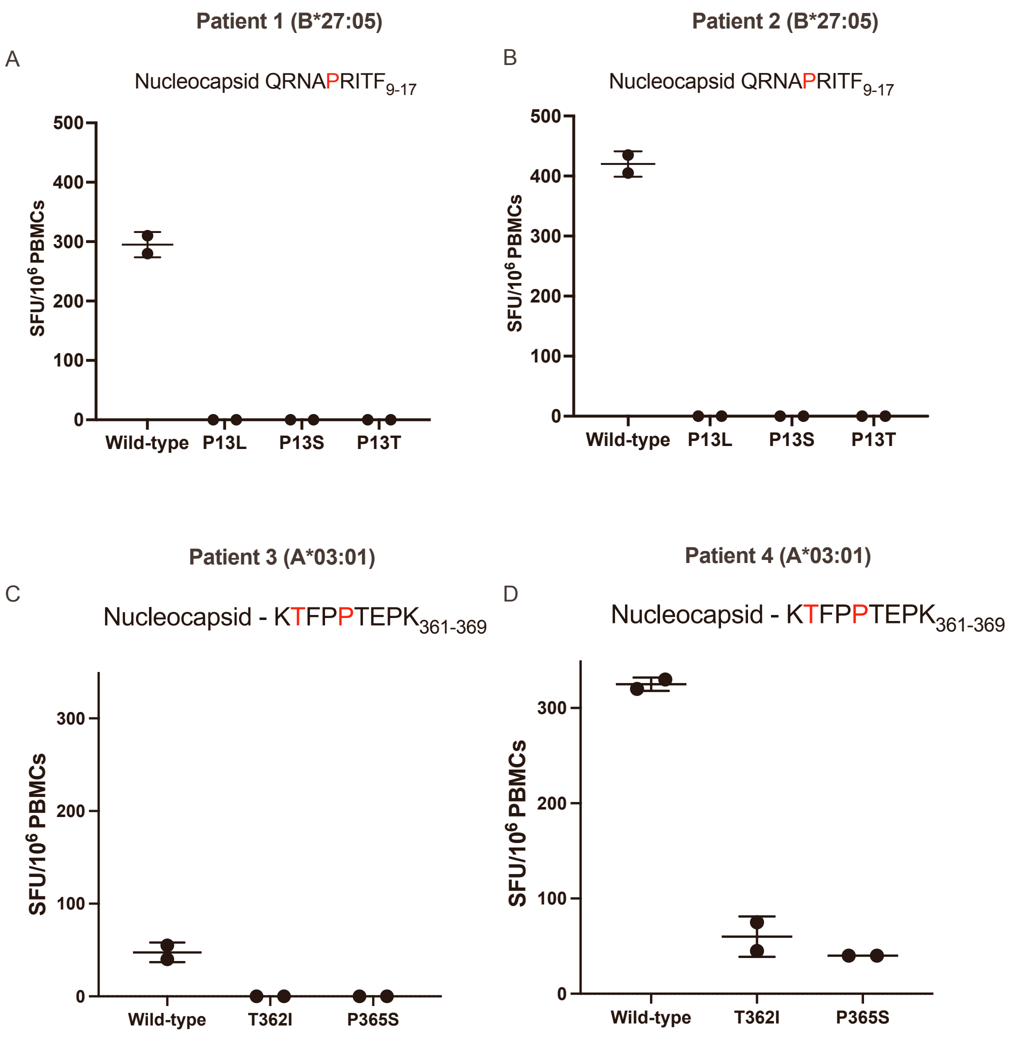

Figure S5. Ex-vivo IFN-γ ELISpot responses to wild-type and variant peptides, related to Figure 1. A and B. Peripheral blood mononuclear cells from two B*27:05 patients tested with the wild-type peptide QRNAPRITF$_{9\text{-}17}$ and mutants P13L, P13S and P13T. C and D. Peripheral blood mononuclear cells from two A*03:01 patients tested with wild-type peptide KTFPPTEPK$_{361\text{-}369}$ and mutant T362I and P365S.

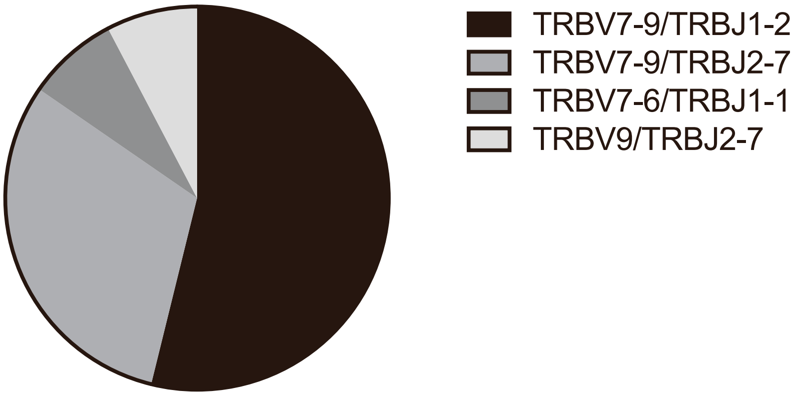

Figure S6. T-cell receptor (TCR) sequence data from a CD8+ polyclonal T-cell line specific for the B*27:05-restricted nucleocapsid epitope QRNAPRITF$_{9\text{-}17}$, related to Figure 2. Data are the proportion of all sequences with each TCR clonotype in a polyclonal line from one donor, where complete loss of peptide recognition was seen with mutants P13L, P13S and P13T (position 5).

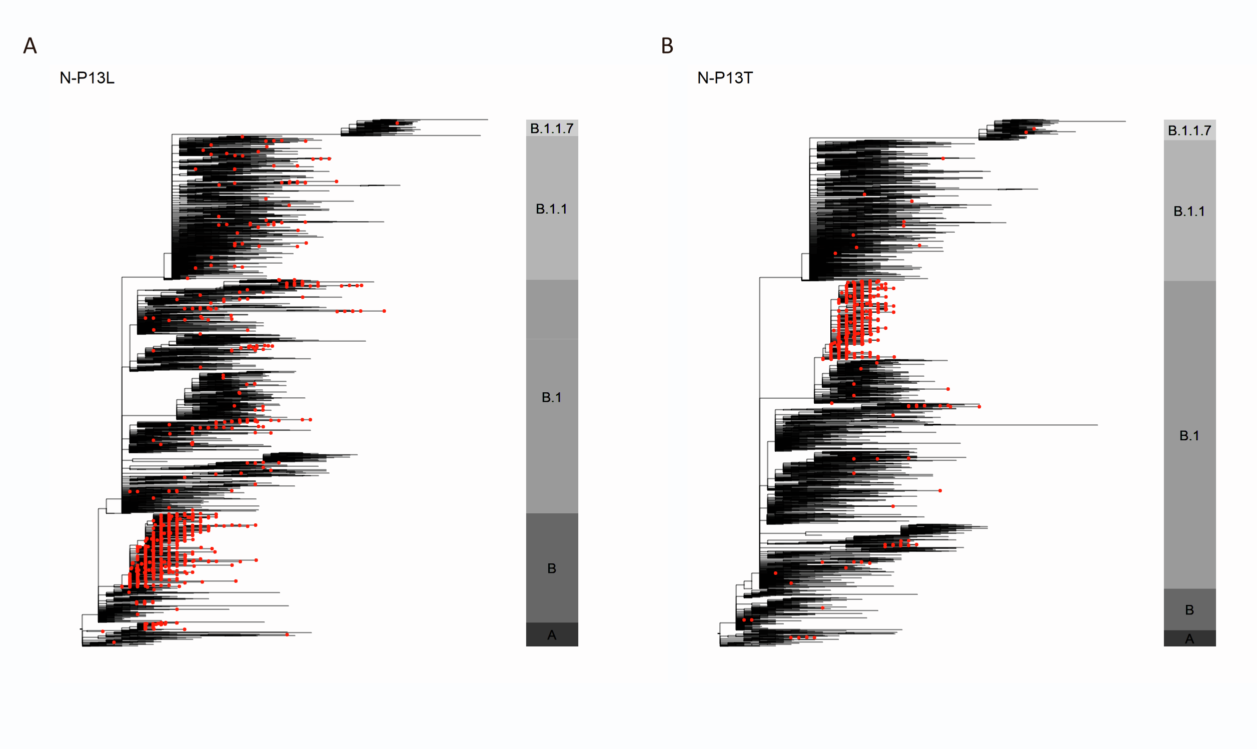

Figure S7. A representative phylogenetic trees of global SARS-CoV-2 genomes depicting the presence of P13L (A) and P13T (B) variants in the nucleocapsid QRNAPRITF$_{9\text{-}17}$ CD8+ T-cell epitope, related to Figure 3. Phylogenies represent all available P13L/P13T sequences (red tips), along with a selection of non-P13L/P13T sequences, which were subsampled for visualisation purposes. The bar to the right of each phylogeny is annotated by main ancestral lineages only and not each individual PANGO lineage that viruses belong to. The grapevine pipeline (https://github.com/COG-UK/grapevine) was used for generating the phylogeny based on all data available on GISAID and COG-UK up until 16th of February 2021.

Epitope	ORF	Amino acid residues	HLA restriction	Variant	Loss of T-cell response	Predicted binding affinity to MHC (IC50 nM)	Difference in predicted binding from wild-type epitope	Binding level
FTSDYYQLY	3a	207 - 215	A*01:01	-	-	5.36	-	Strong
FTSDYYKLY				Q213K	Yes	21.36	3.99-fold decrease	Strong
QRNAPRITF	N	9 - 17	B*27:05	-	-	509.43	-	Strong
QRNALRITF				P13L	Yes	83.06	6.1-fold increase	Strong
QRNASRITF				P13S	Yes	346.15	1.47-fold increase	Strong
QRNATRITF				P13T	Yes	229.47	2.22-fold increase	Strong
MEVTPSGTWL	N	322 - 331	B*40:01	-	-	33.85	-	Strong
MEVIPSGTWL				T325I	Partial	34.59	Neutral	Strong
KTFPPTEPK	N	361 - 369	A*03:01	-	-	19.39	-	Strong
KIFPPTEPK				T362I	Yes	16.20	Neutral	Strong
KTFPSTEPK				P365S	Yes	24.96	Neutral	Strong
KTFPPTEPK			A*11:01	-	-	8.51	-	Strong
KIFPPTEPK				T362I	Yes	11.12	Neutral	Strong
KTFPSTEPK				P365S	Yes	7.73	Neutral	Strong

Table S4. Binding predictions of wild-type and mutant epitopes to Major Histocompatibility Complex (MHC) alleles, related to Figure 1. Predictions derived from NetMHCpan 4.1 (http://www.cbs.dtu.dk/services/NetMHCpan/). ORF = open reading frame, HLA = human leukocyte antigen.
