# Supplementary figures and images for: The impact of viral mutations on recognition by SARS-CoV-2 specific T cells
Source: iScience. 2021 Oct 28;24(11):103353. doi: 10.1016/j.isci.2021.103353 (PMC8552693; doi:10.1016/j.isci.2021.103353)

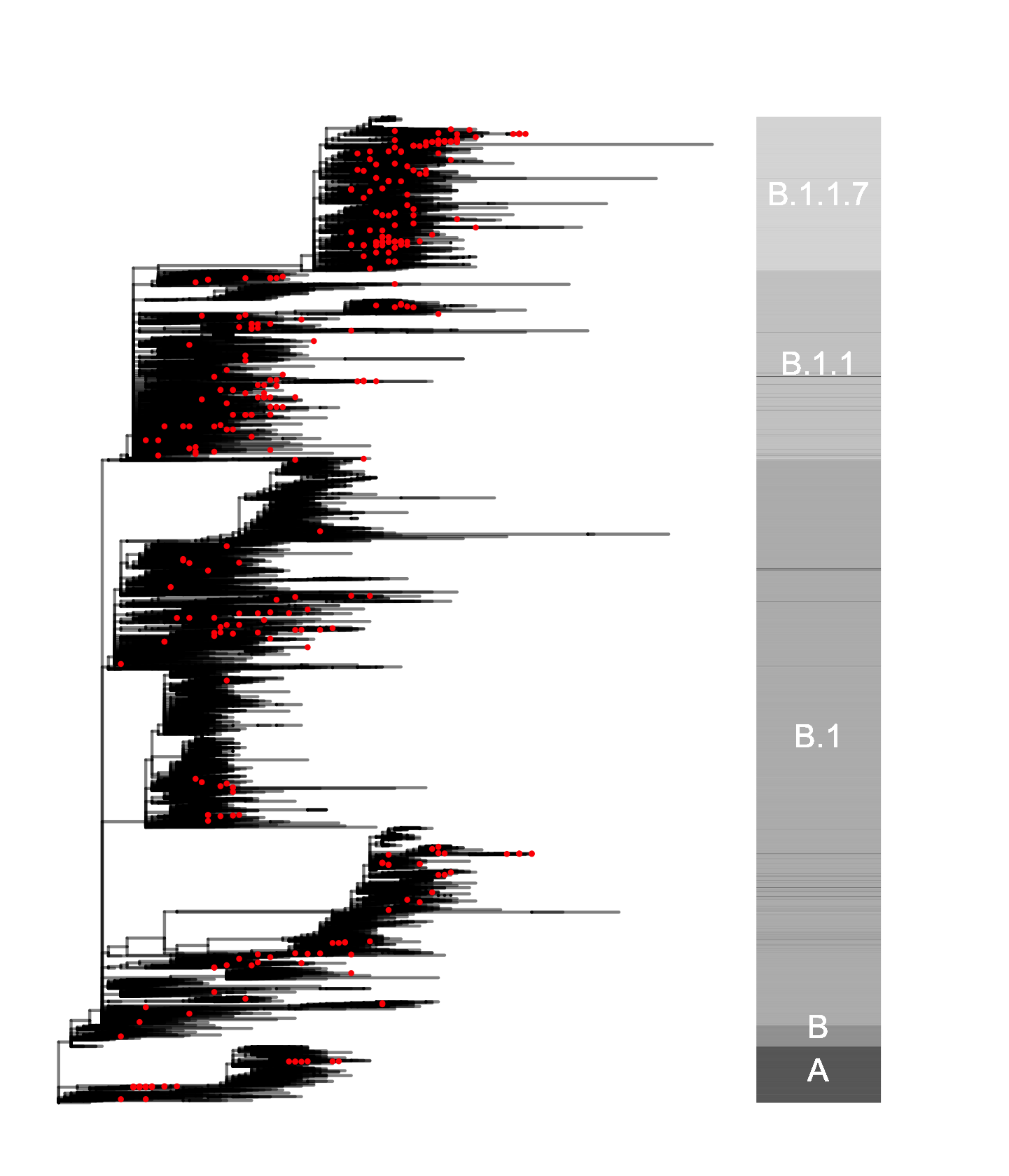

Supplement: Data S2. Variant_prevalence — R code and input data used for plotting the prevalence of variants within T cell epitopes over time [file mmc6.zip › Tree_visualisation/output/ORF3A_Q213K_tree.png]

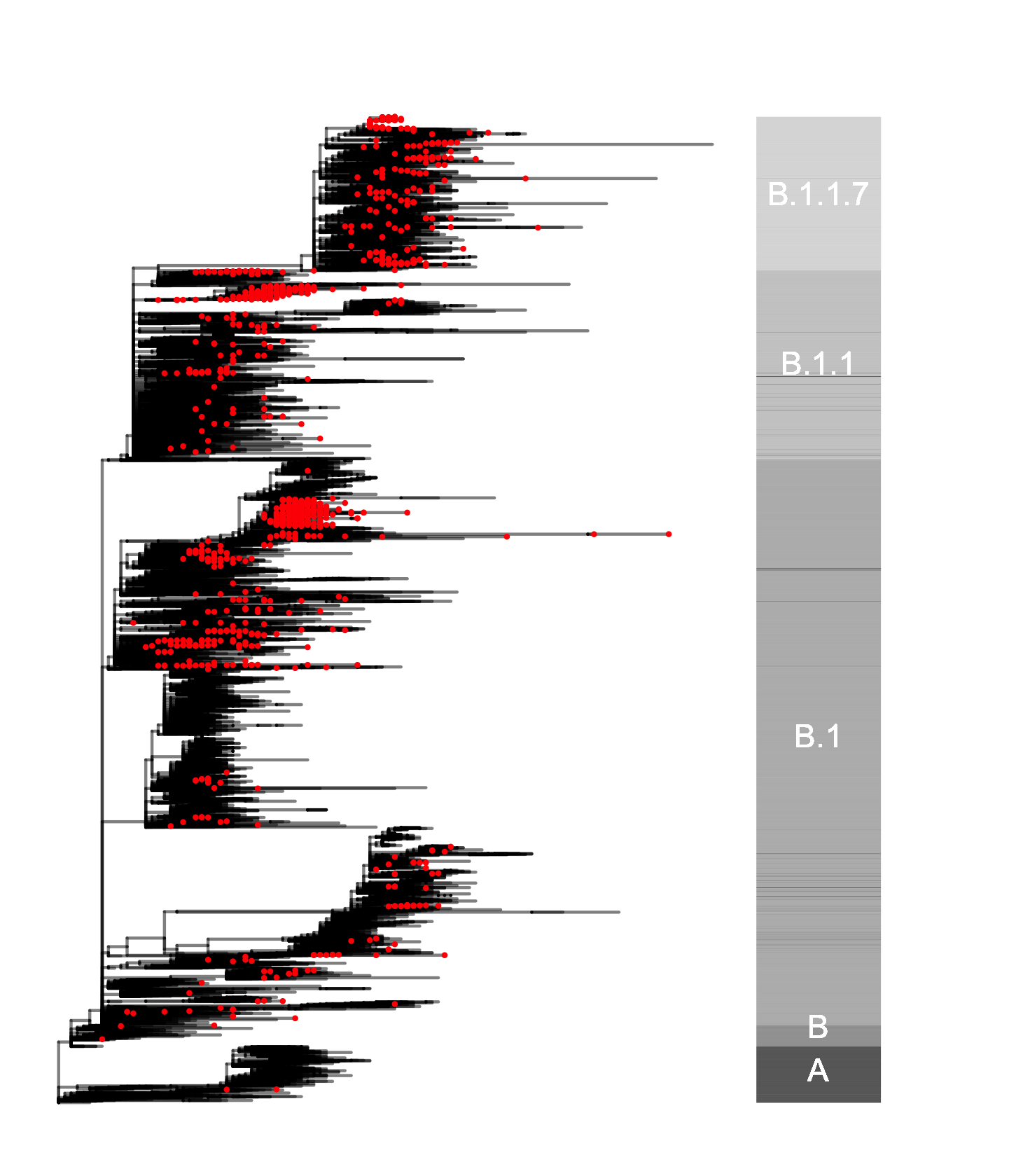

Supplement: Data S2. Variant_prevalence — R code and input data used for plotting the prevalence of variants within T cell epitopes over time [file mmc6.zip › Tree_visualisation/output/N_P13S_tree.png]

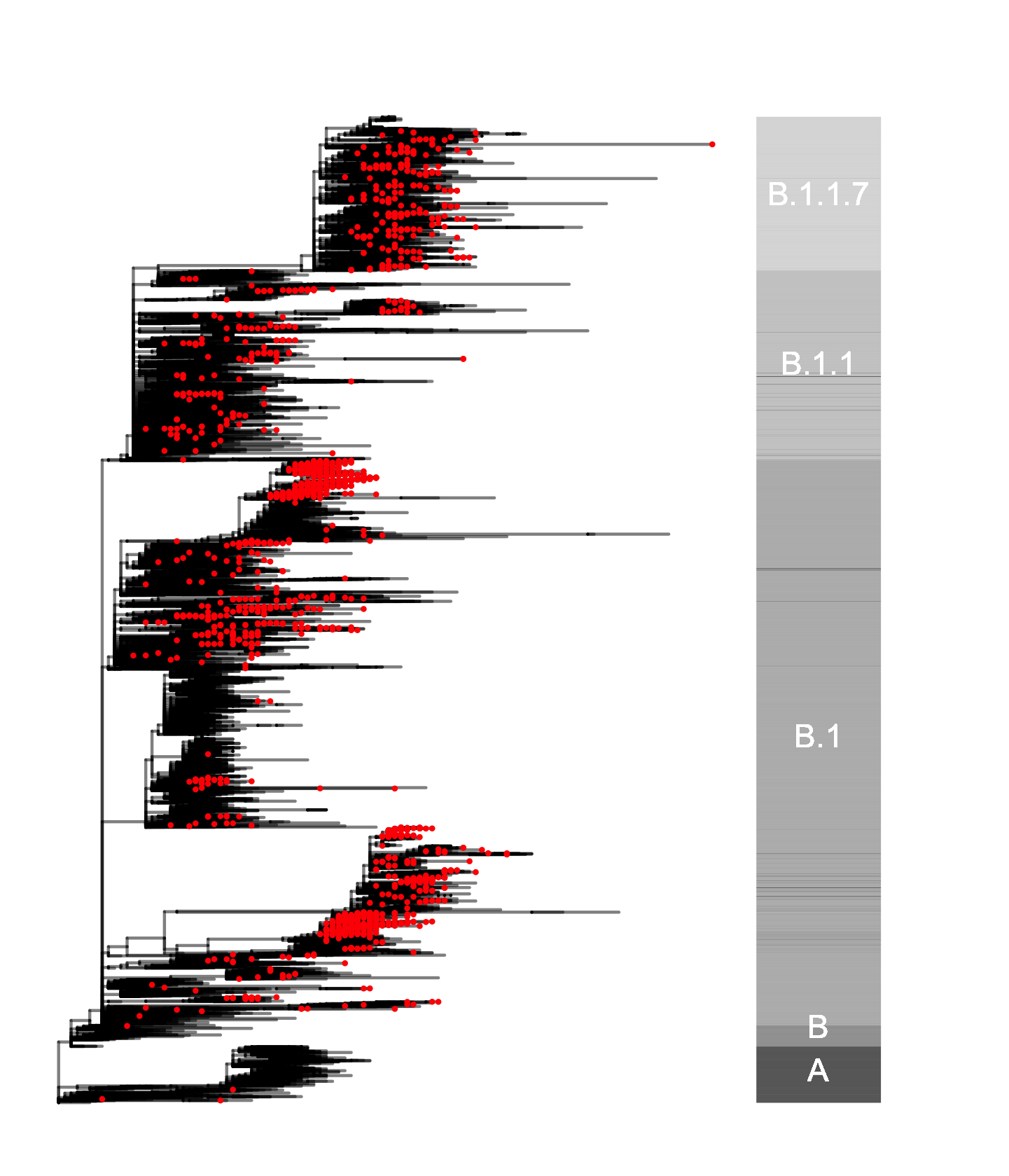

Supplement: Data S2. Variant_prevalence — R code and input data used for plotting the prevalence of variants within T cell epitopes over time [file mmc6.zip › Tree_visualisation/output/N_T362I_tree.png]

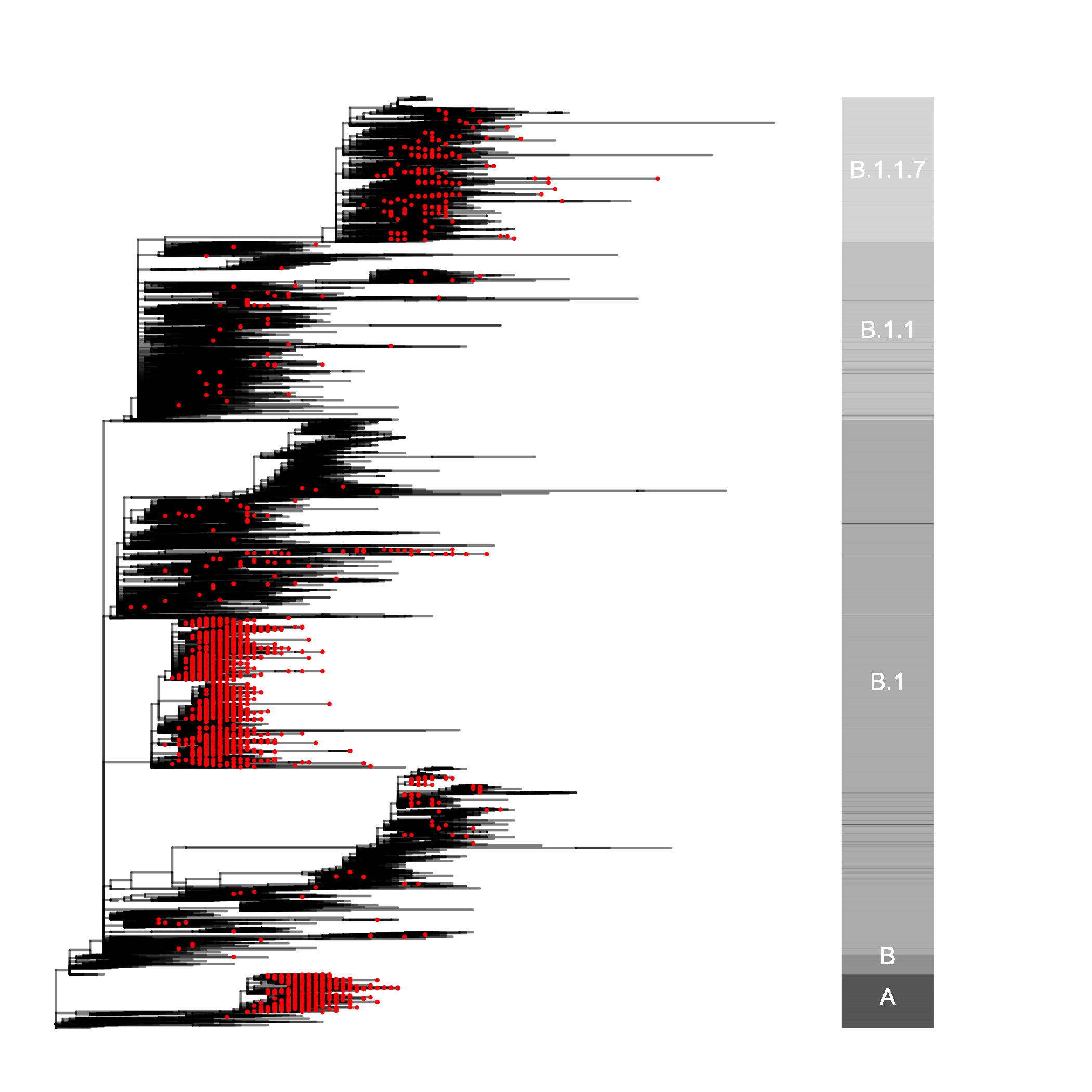

Supplement: Data S2. Variant_prevalence — R code and input data used for plotting the prevalence of variants within T cell epitopes over time [file mmc6.zip › Tree_visualisation/output/N_P365S_tree.png]

T cell epitope variants over time

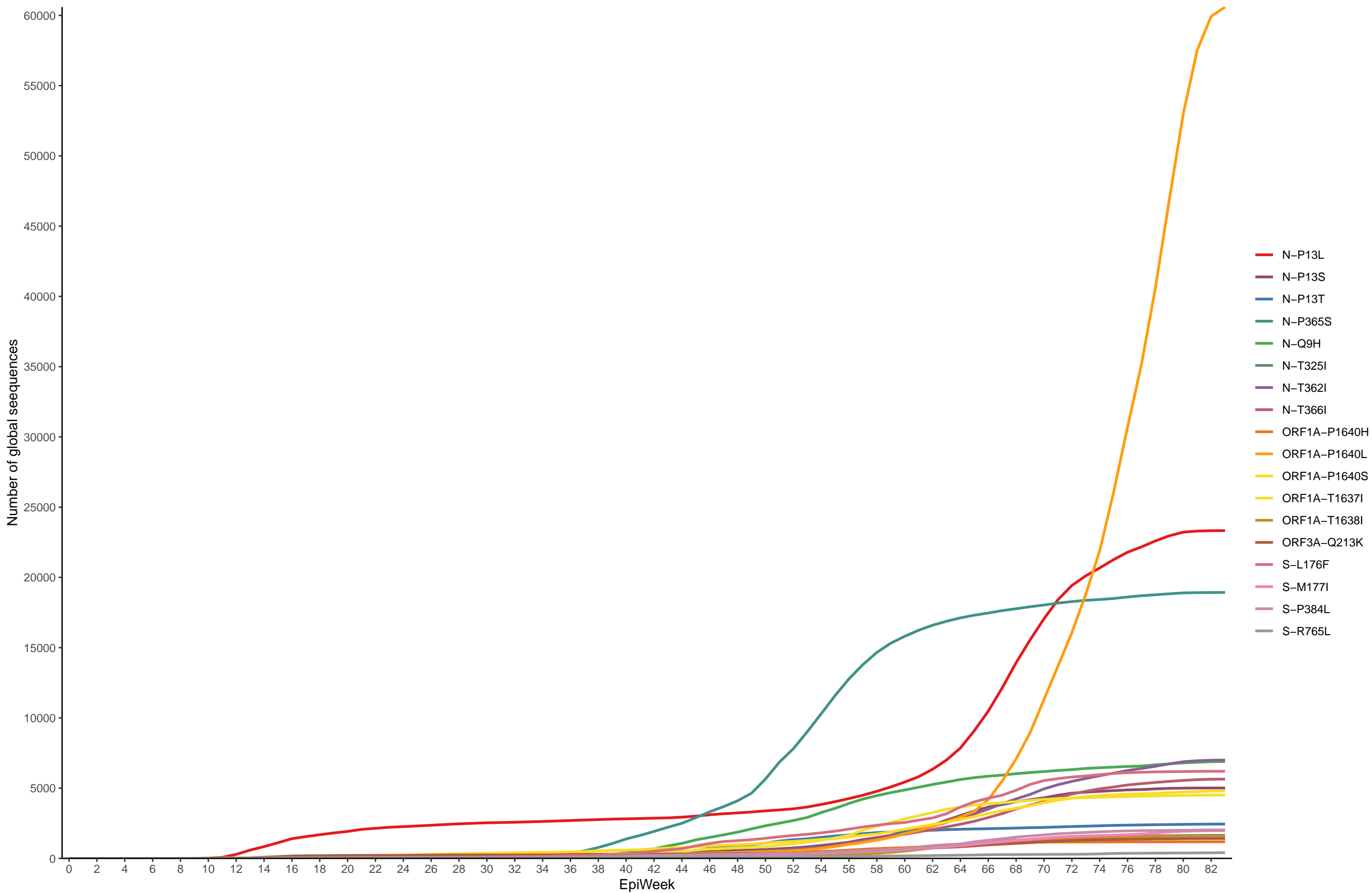

Supplement: Data S3. Tree_visualization — R code and putput data used for plotting representative global phylogenies, highlighting the presence of the variant of interest [file mmc7.zip › Variant_prevalence/output/iScience_mutation_prevalence.pdf]
